# Supplementary material for: Gastrointestinal adverse events associated with tirzepatide: A bibliometric and pharmacovigilance analysis
Source: PLoS One. 2026 Mar 27;21(3):e0344289. doi: 10.1371/journal.pone.0344289 (PMC13028446; doi:10.1371/journal.pone.0344289)

## **S1 Fig. The main steps in the processing of the FAERS database.** Demo, demographics; DRUG, drug; REAC, reaction; PS, Primary suspect; GIAEs: gastrointestinal adverse reactions; ^a^To identify the "best primaryid" for cases, follow these steps: For reports with the same caseid, choose the one with the most recent FDA_DT. If both caseid and FDA_DT are identical, select the report with the highest primaryid. From Q1 2019, a new text file lists deleted cases. The FDA or manufacturers may delete cases for reasons like consolidation. Reports in the delete file are excluded.


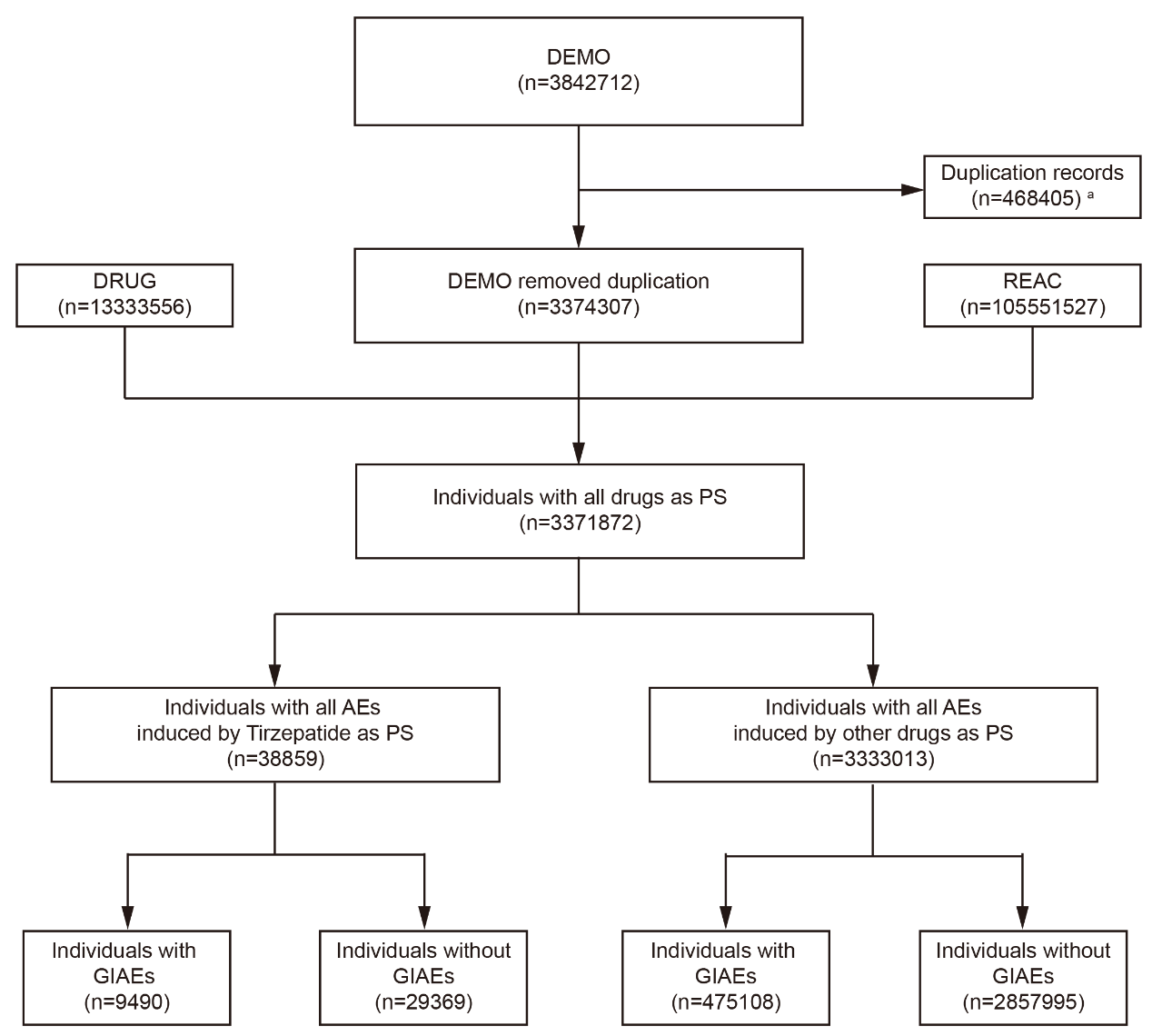

Supplement: S1 Fig — (DOCX) [file pone.0344289.s001.docx]
