# Supplementary material for: Gastrointestinal adverse events associated with tirzepatide: A bibliometric and pharmacovigilance analysis
Source: PLoS One. 2026 Mar 27;21(3):e0344289. doi: 10.1371/journal.pone.0344289 (PMC13028446; doi:10.1371/journal.pone.0344289)
Supplement: S1 Table — (DOCX) [file pone.0344289.s002.docx]

## **S1 Table. Web of Science search strategy**

| **Database** | **Search strategy** | **Number of articles** |
| --- | --- | --- |
| Web of science  (~ -18/11/2024) | #1 TS= (Tirzepatide OR LY3298176 OR zepbound OR Mounjaro)  #2 TS= (Side Effects OR Side Effect OR Adverse Reaction OR Adverse Reactions OR Drug Reaction OR Drug Reactions OR Drug Event OR Drug Events OR Toxicity OR Toxicities OR Drug Reaction OR Drug Reactions)  #3 #2 AND #1 | 945  1615461  110 |
