# Supplementary material for: Gastrointestinal adverse events associated with tirzepatide: A bibliometric and pharmacovigilance analysis
Source: PLoS One. 2026 Mar 27;21(3):e0344289. doi: 10.1371/journal.pone.0344289 (PMC13028446; doi:10.1371/journal.pone.0344289)
Supplement: S2 Table — (DOCX) [file pone.0344289.s003.docx]

## **S2 Table. MedDRA 27 search terms for gastrointestinal disorders**

| **SOC** | **Preferred terms (1064)** |
| --- | --- |
| gastrointestinal disorder | "DIARRHOEA, VOMITING, DYSPEPSIA, ABDOMINAL PAIN, ANORECTAL DISCOMFORT, PROCTALGIA, RECTAL HAEMORRHAGE, DRY MOUTH, SWOLLEN TONGUE, GASTRIC DISORDER, ERUCTATION, NAUSEA, TOOTH DISORDER, ILEUS PARALYTIC, ABDOMINAL DISTENSION, ABDOMINAL DISCOMFORT, DENTAL PULP DISORDER, GASTROINTESTINAL TOXICITY, ABDOMINAL PAIN LOWER, LIP DRY, ODYNOPHAGIA, OESOPHAGEAL PAIN, GASTROOESOPHAGEAL REFLUX DISEASE, GASTRIC HAEMORRHAGE, OESOPHAGEAL PERFORATION, CONSTIPATION, ABDOMINAL PAIN UPPER, PANCREATIC DISORDER, COELIAC DISEASE, TOOTH LOSS, LIP DISCOLOURATION, PANCREATITIS, GINGIVAL RECESSION, FAECES PALE, TOOTHACHE, HAEMATOCHEZIA, MOUTH HAEMORRHAGE, PAROTID GLAND ENLARGEMENT, SALIVARY HYPERSECRETION, INTESTINAL OBSTRUCTION, FAECAL INCONTINENCE, PANCREATITIS ACUTE, GASTROINTESTINAL HAEMORRHAGE, DUODENAL ULCER, GASTRITIS EROSIVE, MELAENA, FLATULENCE, SENSITIVITY OF TEETH, DYSPHAGIA, APHTHOUS STOMATITIS, GINGIVAL BLEEDING, GINGIVAL ERYTHEMA, GINGIVAL HYPERPLASIA, GINGIVAL INFLAMMATION, GINGIVAL PAIN, GASTROINTESTINAL OBSTRUCTION, TOOTH DEVELOPMENT DISORDER, APPENDIX DISORDER, STOMATITIS, TEETH BRITTLE, OESOPHAGITIS, CROHN'S DISEASE, TONGUE DISORDER, COLITIS ULCERATIVE, INTESTINAL HAEMORRHAGE, LOWER GASTROINTESTINAL HAEMORRHAGE, LARGE INTESTINE POLYP, DIVERTICULUM, ABDOMINAL TENDERNESS, ORAL MUCOSAL ERYTHEMA, ORAL PAIN, CHAPPED LIPS, LIP SWELLING, ORAL MUCOSAL BLISTERING, INGUINAL HERNIA, UMBILICAL HERNIA, CHRONIC GASTRITIS, LIP BLISTER, HYPOAESTHESIA ORAL, OEDEMATOUS PANCREATITIS, HAEMORRHOIDS, GASTROINTESTINAL DISORDER, INTESTINAL ISCHAEMIA, RETCHING, MOUTH ULCERATION, SUBILEUS, POUCHITIS, COLITIS, SMALL INTESTINAL STENOSIS, GLOSSODYNIA, PARAESTHESIA ORAL, INTRA-ABDOMINAL HAEMATOMA, INTRA-ABDOMINAL HAEMORRHAGE, LIP OEDEMA, ASCITES, ABDOMINAL WALL HAEMATOMA, EPIGASTRIC DISCOMFORT, ILEAL STENOSIS, ENTEROCOLITIS, RECTAL PROLAPSE, FAECES DISCOLOURED, ORAL DISCOMFORT, GASTROINTESTINAL PAIN, GASTROINTESTINAL TRACT IRRITATION, OESOPHAGEAL ULCER, TONGUE ULCERATION, GASTROINTESTINAL INFLAMMATION, INTESTINAL MUCOSAL HYPERTROPHY, INTESTINAL STENOSIS, TONGUE OEDEMA, APHAGIA, TOOTH DISCOLOURATION, IMPAIRED GASTRIC EMPTYING, UPPER GASTROINTESTINAL HAEMORRHAGE, TONGUE BLISTERING, GASTROINTESTINAL PERFORATION, LEUKOPLAKIA ORAL, REGURGITATION, ATROPHIC GLOSSITIS, HAEMATEMESIS, OESOPHAGEAL RUPTURE, ENLARGED UVULA, SMALL INTESTINAL HAEMORRHAGE, HIATUS HERNIA, PEPTIC ULCER, DEFAECATION URGENCY, BREATH ODOUR, GASTRITIS, GASTROINTESTINAL MOTILITY DISORDER, VOLVULUS, INTESTINAL PERFORATION, NECROTISING COLITIS, SMALL INTESTINAL OBSTRUCTION, TONGUE DRY, GINGIVAL SWELLING, LOOSE TOOTH, ORAL DISORDER, GASTRIC ULCER, FOOD POISONING, ABDOMINAL SYMPTOM, VOMITING PROJECTILE, ENTEROVESICAL FISTULA, DIARRHOEA HAEMORRHAGIC, DIVERTICULUM INTESTINAL HAEMORRHAGIC, RECTAL TENESMUS, HAEMORRHOIDAL HAEMORRHAGE, OESOPHAGEAL SPASM, RETROPERITONEAL HAEMORRHAGE, GASTROINTESTINAL SOUNDS ABNORMAL, PERITONEAL CLOUDY EFFLUENT, IRRITABLE BOWEL SYNDROME, INFLAMMATORY BOWEL DISEASE, EROSIVE OESOPHAGITIS, DIVERTICULAR PERFORATION, ABDOMINAL HERNIA, LIP ULCERATION, OEDEMA MOUTH, CHEILITIS, LIP EROSION, LIP HAEMORRHAGE, ORAL MUCOSA EROSION, STOMATITIS NECROTISING, TONGUE COATED, GASTROINTESTINAL MUCOSAL EXFOLIATION, ORAL MUCOSAL EXFOLIATION, ABDOMINAL WALL HAEMORRHAGE, FREQUENT BOWEL MOVEMENTS, ANAL SKIN TAGS, ABDOMINAL ADHESIONS, LIP DISORDER, GASTRIC DILATATION, DENTAL CARIES, COLITIS MICROSCOPIC, PANCREATITIS CHRONIC, BOWEL MOVEMENT IRREGULARITY, ENTEROCOLITIS HAEMORRHAGIC, ANAL HAEMORRHAGE, TONGUE DISCOLOURATION, ABNORMAL FAECES, COLITIS ISCHAEMIC, ANAL FISTULA, GASTROINTESTINAL ULCER, OESOPHAGEAL OEDEMA, ENTERITIS, ANORECTAL DISORDER, INTESTINAL ULCER, DUODENAL ULCER HAEMORRHAGE, MOUTH SWELLING, PALATAL OEDEMA, GASTRITIS HAEMORRHAGIC, SALIVA ALTERED, FAECALOMA, MESENTERIC ARTERY THROMBOSIS, ORAL PRURITUS, DUODENAL POLYP, PERITONEAL HAEMORRHAGE, FAECALITH, ILEUS, INTUSSUSCEPTION, LARGE INTESTINAL HAEMORRHAGE, ULTRAFILTRATION FAILURE, LARGE INTESTINE PERFORATION, GASTROENTERITIS EOSINOPHILIC, GINGIVAL DISCOLOURATION, GINGIVAL DISORDER, ENTEROCUTANEOUS FISTULA, ILEAL PERFORATION, HYPERCHLORHYDRIA, PANCREATIC MASS, TOOTH EROSION, LARGE INTESTINAL OBSTRUCTION, CHANGE OF BOWEL HABIT, RECTAL OBSTRUCTION, RETROPERITONEAL FIBROSIS, GASTRIC PERFORATION, FAECES SOFT, INTESTINAL PROLAPSE, MEGACOLON, GASTROINTESTINAL MUCOSAL NECROSIS, LARGE INTESTINAL STENOSIS, OESOPHAGEAL STENOSIS, INTESTINAL FISTULA, LOCALISED INTRAABDOMINAL FLUID COLLECTION, ANAL STENOSIS, HAEMORRHAGIC EROSIVE GASTRITIS, GASTRIC ULCER PERFORATION, INTESTINAL FIBROSIS, GLOSSITIS, FUNCTIONAL GASTROINTESTINAL DISORDER, OESOPHAGEAL DISORDER, NONINFECTIOUS PERITONITIS, ACUTE ABDOMEN, SMALL INTESTINAL PERFORATION, RECTAL ULCER, OESOPHAGEAL ULCER HAEMORRHAGE, INTESTINAL POLYP, GASTROINTESTINAL STENOSIS, GASTROINTESTINAL EROSION, MALABSORPTION, AUTOIMMUNE PANCREATITIS, OESOPHAGEAL COMPRESSION, PANCREATITIS RELAPSING, MESENTERIC VASCULAR OCCLUSION, DUODENAL PERFORATION, JEJUNAL PERFORATION, INTESTINAL MASS, GASTROINTESTINAL NECROSIS, PNEUMOPERITONEUM, GASTRIC ANTRAL VASCULAR ECTASIA, PANCREATIC CYST, OESOPHAGEAL OBSTRUCTION, BARRETT'S OESOPHAGUS, DYSBACTERIOSIS, GASTRIC ULCER HAEMORRHAGE, ABDOMINAL MASS, BEZOAR, PANCREATIC DUCT STENOSIS, GINGIVAL ULCERATION, RECTAL FISSURE, TOOTH IMPACTED, MALIGNANT ASCITES, SMALL INTESTINE ULCER, APHTHOUS ULCER, FAECES HARD, LIP EXFOLIATION, PANCREATIC DUCT DILATATION, RECTAL DISCHARGE, PERIODONTAL DISEASE, RECTAL POLYP, OESOPHAGEAL HAEMORRHAGE, GASTRIC POLYPS, LIP PRURITUS, MUCOUS STOOLS, POOR DENTAL CONDITION, STOMACH MASS, STRAWBERRY TONGUE, PERITONEAL DISORDER, INFREQUENT BOWEL MOVEMENTS, ANAL INFLAMMATION, VARICES OESOPHAGEAL, GASTROINTESTINAL FISTULA, PANCREATITIS HAEMORRHAGIC, ANAL FISSURE, LARGE INTESTINAL ULCER, INTESTINAL DILATATION, GASTRIC VARICES HAEMORRHAGE, THROMBOSIS MESENTERIC VESSEL, GINGIVAL HYPERTROPHY, DIAPHRAGMATIC HERNIA, OESOPHAGEAL IRRITATION, TONGUE EXFOLIATION, ABDOMINAL RIGIDITY, ABDOMINAL COMPARTMENT SYNDROME, PANCREATITIS NECROTISING, EOSINOPHILIC COLITIS, EOSINOPHILIC OESOPHAGITIS, PROTRUSION TONGUE, GASTROINTESTINAL ULCER HAEMORRHAGE, INTESTINAL CYST, MESENTERIC VEIN THROMBOSIS, PORTAL HYPERTENSIVE GASTROPATHY, DUODENITIS, ANAL INCONTINENCE, POST GASTRIC SURGERY SYNDROME, OESOPHAGEAL ACHALASIA, LIP PAIN, DIVERTICULUM INTESTINAL, NEUTROPENIC COLITIS, DENTAL DISCOMFORT, GINGIVITIS ULCERATIVE, OBSTRUCTION GASTRIC, PANCREATIC STEATOSIS, HAEMORRHAGIC ASCITES, TONGUE MOVEMENT DISTURBANCE, NECROTISING ENTEROCOLITIS NEONATAL, PANCREATOLITHIASIS, ENAMEL ANOMALY, PANCREATIC ATROPHY, DOUGLAS' POUCH MASS, GINGIVAL BLISTER, GASTROINTESTINAL HYPOMOTILITY, PANCREATIC PSEUDOCYST, PROTEIN-LOSING GASTROENTEROPATHY, TONGUE ERUPTION, PALATAL DISORDER, TONGUE HAEMORRHAGE, MESENTERIC HAEMATOMA, PROCTITIS, BLOODY PERITONEAL EFFLUENT, COLONIC PSEUDO-OBSTRUCTION, INTESTINAL HAEMATOMA, ACQUIRED OESOPHAGEAL WEB, REFLUX GASTRITIS, TONGUE SPASM, OESOPHAGEAL DISCOMFORT, GASTROINTESTINAL MUCOSAL DISORDER, SPRUE-LIKE ENTEROPATHY, ANAL PRURITUS, EROSIVE DUODENITIS, VASCULITIS GASTROINTESTINAL, RETROPERITONEAL HAEMATOMA, SALIVARY GLAND ENLARGEMENT, ANAL ULCER, ORAL MUCOSAL ERUPTION, PROCTOPARALYSIS, INCARCERATED INGUINAL HERNIA, ILEUS SPASTIC, RETROPERITONEAL MASS, ILEAL ULCER, DUODENITIS HAEMORRHAGIC, VISCERAL ARTERIAL ISCHAEMIA, HYPOAESTHESIA TEETH, ORAL PAPULE, ACQUIRED MACROGLOSSIA, HYPERTROPHY OF TONGUE PAPILLAE, GASTRIC FISTULA, NEUROGENIC BOWEL, MALLORY-WEISS SYNDROME, PANCREATIC INSUFFICIENCY, INTESTINAL VILLI ATROPHY, PNEUMATOSIS INTESTINALIS, NECROTISING OESOPHAGITIS, OESOPHAGITIS HAEMORRHAGIC, OESOPHAGITIS ULCERATIVE, DENTAL PLAQUE, GASTROINTESTINAL ISCHAEMIA, HAEMOPERITONEUM, PEPTIC ULCER PERFORATION, GASTROINTESTINAL ANGIODYSPLASIA HAEMORRHAGIC, GASTRIC MUCOSAL LESION, GASTROINTESTINAL OEDEMA, PANCREATIC ENLARGEMENT, PEPTIC ULCER HAEMORRHAGE, OESOPHAGEAL VARICES HAEMORRHAGE, TOOTH MALFORMATION, COLON DYSPLASIA, OESOPHAGEAL POLYP, OESOPHAGEAL FOOD IMPACTION, GASTROINTESTINAL ANGIODYSPLASIA, DUMPING SYNDROME, TOOTH DEMINERALISATION, INTESTINAL INFARCTION, DENTAL NECROSIS, MESENTERIC ARTERIAL OCCLUSION, AEROPHAGIA, MESENTERIC PANNICULITIS, MECHANICAL ILEUS, COLONIC FISTULA, OESOPHAGEAL MOTILITY DISORDER, SALIVARY GLAND PAIN, RECTAL PERFORATION, ORAL LICHEN PLANUS, PANCREATIC DUCT OBSTRUCTION, GASTROLITHIASIS, TOOTH CROWDING, DIARRHOEA NEONATAL, ENTEROCOLONIC FISTULA, BURNING MOUTH SYNDROME, SHORT-BOWEL SYNDROME, OESOPHAGEAL MUCOSAL DISSECTION, OESOPHAGEAL MUCOSA ERYTHEMA, ORAL MUCOSAL DISCOLOURATION, ANAL SPHINCTER ATONY, ANAL PARAESTHESIA, OESOPHAGEAL DYSPLASIA, DUODENAL FISTULA, TONGUE PRURITUS, GASTROINTESTINAL WALL THICKENING, GINGIVAL ATROPHY, STEATORRHOEA, PANCREATIC NECROSIS, OESOPHAGEAL FISTULA, UVULITIS, VOLVULUS OF SMALL BOWEL, DUODENAL STENOSIS, DENTAL ALVEOLAR ANOMALY, GASTROINTESTINAL TRACT MUCOSAL DISCOLOURATION, PAINFUL DEFAECATION, FAECAL VOMITING, INCARCERATED UMBILICAL HERNIA, COELIAC ARTERY STENOSIS, OMENTAL INFARCTION, PANCREATIC FIBROSIS, GASTROINTESTINAL TRACT MUCOSAL PIGMENTATION, PERITONEAL ADHESIONS, PERITONEAL PERFORATION, HERNIAL EVENTRATION, POST-TUSSIVE VOMITING, OESOPHAGEAL HYPOMOTILITY, ABDOMINAL WALL DISORDER, SMALL INTESTINE POLYP, GLOSSOPTOSIS, GASTRIC MUCOSA ERYTHEMA, GASTRIC VARICES, DISTAL INTESTINAL OBSTRUCTION SYNDROME, RECTAL STENOSIS, ANAL SPASM, ANAL SPHINCTER HYPERTONIA, DUODENAL ULCER PERFORATION, PHARYNGO-OESOPHAGEAL DIVERTICULUM, MESENTERIC ARTERY EMBOLISM, TONGUE DISCOMFORT, ORAL CAVITY FISTULA, GASTRIC CYST, SALIVARY GLAND CYST, DIABETIC GASTROPARESIS, GASTROINTESTINAL MELANOSIS, GASTROINTESTINAL HYPERMOTILITY, ANOVULVAR FISTULA, PANCREATIC FISTULA, ENTEROCELE, GASTROINTESTINAL POLYP HAEMORRHAGE, NONINFECTIVE GINGIVITIS, PERIANAL ERYTHEMA, TOOTH RESORPTION, RETROPERITONEUM CYST, TOOTH PULP HAEMORRHAGE, PERITONEAL CYST, PORTAL VENOUS GAS, GASTRODUODENAL ULCER, GASTROOESOPHAGEAL SPHINCTER INSUFFICIENCY, ANAL PROLAPSE, MESENTERIC VASCULAR INSUFFICIENCY, ALLERGIC COLITIS, PYLORIC SPHINCTER INSUFFICIENCY, GINGIVAL EROSION, ALVEOLAR BONE RESORPTION, MELANOSIS COLI, PANCREATIC CYST RUPTURE, SPIGELIAN HERNIA, DYSCHEZIA, LUMBAR HERNIA, PAROTID GLAND HAEMORRHAGE, OESOPHAGEAL DILATATION, PSEUDOPOLYPOSIS, ANAL EROSION, RECTAL LESION, CYCLIC VOMITING SYNDROME, TOOTH SOCKET HAEMORRHAGE, ALLERGIC GASTROENTERITIS, SCLEROSING ENCAPSULATING PERITONITIS, LUPUS ENTERITIS, INFANTILE SPITTING UP, ALCOHOLIC PANCREATITIS, HAEMORRHOIDS THROMBOSED, PLICATED TONGUE, GINGIVAL OEDEMA, GINGIVAL CYST, ORAL DISCHARGE, SALIVARY GLAND CALCULUS, SMALL INTESTINAL ULCER PERFORATION, APTYALISM, HEYDE'S SYNDROME, CHEILITIS GRANULOMATOSA, ORAL DYSAESTHESIA, BUCCAL MUCOSAL ROUGHENING, PERITONEAL NECROSIS, SMALL BOWEL ANGIOEDEMA, OESOPHAGEAL ATONY, GASTRIC MUCOSAL HYPERTROPHY, RECTAL ULCER HAEMORRHAGE, TONGUE GEOGRAPHIC, GASTRODUODENAL HAEMORRHAGE, RECTAL SPASM, SALIVA DISCOLOURATION, INTESTINAL ULCER PERFORATION, PAPILLA OF VATER STENOSIS, OROANTRAL FISTULA, TOOTH DEPOSIT, ISCHAEMIC ENTERITIS, LARGE INTESTINAL ULCER HAEMORRHAGE, DIEULAFOY'S VASCULAR MALFORMATION, TONGUE HAEMATOMA, GASTRODUODENITIS, ANORECTAL SWELLING, UMBILICAL HERNIA PERFORATION, AORTO-OESOPHAGEAL FISTULA, GINGIVAL POLYP, GASTROINTESTINAL VASCULAR MALFORMATION, MESENTERIC HAEMORRHAGE, ABDOMINAL HERNIA OBSTRUCTIVE, ABDOMINAL WALL CYST, PANCREATIC ENZYME ABNORMALITY, SALIVARY GLAND DISORDER, MALOCCLUSION, SALIVARY GLAND ATROPHY, CHRONIC GASTROINTESTINAL BLEEDING, TONGUE NECROSIS, FEMORAL HERNIA, CARDIOSPASM, PANCREATIC HAEMORRHAGE, EPULIS, INTESTINAL SCARRING, SMALL INTESTINAL ULCER HAEMORRHAGE, ANGULAR CHEILITIS, DIVERTICULUM OESOPHAGEAL, GASTROINTESTINAL WALL THINNING, AORTO-DUODENAL FISTULA, RICHTER'S HERNIA, DUODENAL OBSTRUCTION, DUODENAL VASCULAR ECTASIA, ISCHAEMIC GASTRITIS, NEONATAL INTESTINAL DILATATION, AUTOIMMUNE COLITIS, PROCTITIS ULCERATIVE, RECTOURETHRAL FISTULA, INTESTINAL DIAPHRAGM DISEASE, SPLENIC ARTERY ANEURYSM, PERISTALSIS VISIBLE, APPENDICITIS NONINFECTIVE, PERITONEAL HERNIA, OESOPHAGEAL MASS, VARICOSE VEINS OF ABDOMINAL WALL, PROCTITIS HAEMORRHAGIC, GASTROINTESTINAL TELANGIECTASIA, PANCREATIC CALCIFICATION, INTESTINAL MUCOSAL ATROPHY, INTERNAL HERNIA, BILE ACID MALABSORPTION, ANAL POLYP, APICAL GRANULOMA, JEJUNAL STENOSIS, DISCOLOURED VOMIT, ANAL DILATATION, MECONIUM ILEUS, PALATAL SWELLING, PIGMENTATION LIP, ABDOMINAL HERNIA PERFORATION, INTESTINAL METAPLASIA, SALIVARY DUCT OBSTRUCTION, GASTRIC STENOSIS, PIGMENTATION BUCCAL, APOPTOTIC COLONOPATHY, SALIVARY GLAND MUCOCOELE, ILEAL FISTULA, SALIVARY GLAND MASS, GASTROINTESTINAL ULCER PERFORATION, PERITONEAL HAEMATOMA, MECONIUM CYST, MECONIUM PERITONITIS, HYPERAESTHESIA TEETH, ORAL MUCOSA HAEMATOMA, JEJUNAL ULCER, PERITONITIS SCLEROSING, ENTERITIS LEUKOPENIC, GASTRIC VOLVULUS, GINGIVAL PRURITUS, FISTULA OF SMALL INTESTINE, DENTAL CYST, LYMPHANGIECTASIA INTESTINAL, GASTRIC ILEUS, INTRA-ABDOMINAL FLUID COLLECTION, ORAL TOXICITY, INFANTILE COLIC, GASTRIC HYPERMOTILITY, COATING IN MOUTH, BRUNNER'S GLAND HYPERPLASIA, EPIPLOIC APPENDAGITIS, MESENTERIC ARTERY STENOSIS, ANGINA BULLOSA HAEMORRHAGICA, ANAL ULCER HAEMORRHAGE, MOUTH CYST, DEFAECATION DISORDER, PANCREATIC FAILURE, ABDOMINAL FAT APRON, PERITONEAL LESION, RADICULAR CYST, PERIODONTAL INFLAMMATION, GASTRIC HYPOMOTILITY, ANOGENITAL DYSPLASIA, SALIVARY DUCT INFLAMMATION, OESOPHAGEAL ULCER PERFORATION, JEJUNAL ULCER PERFORATION, PYLORUS DILATATION, TONGUE ERYTHEMA, COLONIC HAEMATOMA, TEETHING, HYPERTROPHIC ANAL PAPILLA, PYLOROSPASM, TONGUE ATROPHY, GASTROINTESTINAL DYSPLASIA, MALACOPLAKIA GASTROINTESTINAL, PNEUMORETROPERITONEUM, INTESTINAL STRANGULATION, ABDOMINAL MIGRAINE, NECROTISING GASTRITIS, INFANTILE VOMITING, TOXIC DILATATION OF INTESTINE, FEMORAL HERNIA INCARCERATED, PERITONEAL FIBROSIS, COLITIS EROSIVE, INGUINAL HERNIA STRANGULATED, NONINFECTIVE SIALOADENITIS, DIABETIC GASTROPATHY, INTESTINAL PSEUDO-OBSTRUCTION, COELIAC ARTERY COMPRESSION SYNDROME, HIATUS HERNIA STRANGULATED, ABDOMINAL INCARCERATED HERNIA, ABDOMINAL WALL MASS, DIVERTICULUM GASTRIC, NARCOTIC BOWEL SYNDROME, VARICOSE VEINS SUBLINGUAL, DYSBIOSIS, INGUINAL HERNIA, OBSTRUCTIVE, GASTROINTESTINAL MUCOSA HYPERAEMIA, GASTROINTESTINAL ANGIECTASIA, FEMORAL HERNIA STRANGULATED, PAROTID DUCT OBSTRUCTION, STRESS ULCER, PANCREATIC INFARCTION, INTESTINAL VARICES, CEMENTO OSSEOUS DYSPLASIA, GINGIVAL DISCOMFORT, ISCHAEMIC PANCREATITIS, DUODENOGASTRIC REFLUX, TOOTH DELAMINATION, LUPUS PANCREATITIS, TONGUE CYST, PELVIC FLOOR DYSSYNERGIA, NEONATAL GASTROINTESTINAL HAEMORRHAGE, APPENDICEAL MUCOCOELE, DYSKINESIA OESOPHAGEAL, TONGUE DYSPLASIA, TONGUE AMYLOIDOSIS, SUPERIOR MESENTERIC ARTERY SYNDROME, LIP ERYTHEMA, INTESTINAL ANGINA, ANORECTAL STENOSIS, GASTROINTESTINAL AMYLOIDOSIS, GASTRIC HYPERTONIA, TRUNCUS COELIACUS THROMBOSIS, PALATAL DYSPLASIA, TONGUE POLYP, ABDOMINAL REBOUND TENDERNESS, MALPOSITIONED TEETH, LARGE INTESTINE EROSION, ACHLORHYDRIA, GASTRIC PROLAPSE, TONGUE PIGMENTATION, ORAL MUCOSAL HYPERTROPHY, MESENTERIC ARTERIOSCLEROSIS, SUBMAXILLARY GLAND ENLARGEMENT, INTESTINAL ANGIOEDEMA, GASTROPTOSIS, STASIS SYNDROME, ANORECTAL VARICES, CHEILOSIS, INTESTINAL CONGESTION, FIBROSING COLONOPATHY, MESENTERIC VENOUS OCCLUSION, SIALOMETAPLASIA, HYPERGASTRINAEMIA, PANCREATIC TOXICITY, DIABETIC GASTROENTEROPATHY, AMALGAM TATTOO, TRAUMATIC OCCLUSION, ABDOMINAL STRANGULATED HERNIA, GASTROINTESTINAL VASCULAR MALFORMATION HAEMORRHAGIC, BILIARY ASCITES, SANDIFER'S SYNDROME, NEONATAL INTESTINAL PERFORATION, GASTROINTESTINAL WALL ABNORMAL, PRESBYOESOPHAGUS, DIVERTICULITIS OESOPHAGEAL, DIVERTICULAR FISTULA, DIABETIC ENTEROPATHY, STOMATITIS HAEMORRHAGIC, ORAL SUBMUCOSAL FIBROSIS, GINGIVAL HYPOPLASIA, GIANT CELL EPULIS, GASTRIC DYSPLASIA, ACETONAEMIC VOMITING, ORAL BLOOD BLISTER, MESENTERIC PHLEBOSCLEROSIS, ANKYLOGLOSSIA ACQUIRED, LIP HAEMATOMA, ISCHIORECTAL HERNIA, HAEMORRHAGIC NECROTIC PANCREATITIS, UMBILICAL HERNIA, OBSTRUCTIVE, PARESIS ANAL SPHINCTER, ILEAL ULCER PERFORATION, ANAL HYPOAESTHESIA, MESENTERITIS, INCARCERATED HIATUS HERNIA, APPENDICOLITH, OESOPHAGEAL MUCOSAL TEAR, GASTRIC XANTHOMA, PELVIC FLOOR DYSFUNCTION, ULCERATIVE GASTRITIS, MASTOCYTIC ENTEROCOLITIS, IMMUNE-MEDIATED ENTEROCOLITIS, LEVATOR SYNDROME, GASTRIC ATONY, LYMPHOID HYPERPLASIA OF INTESTINE, MALIGNANT DYSPHAGIA, ORAL HYPERAESTHESIA, ANORECTAL VARICES HAEMORRHAGE, ENCAPSULATING PERITONEAL SCLEROSIS, CASCADE STOMACH, GASTROSPLENIC FISTULA, FIXED BOWEL LOOP, MECONIUM PLUG SYNDROME, ECTOPIC GASTRIC MUCOSA, TRICHOGLOSSIA, ULCERATIVE DUODENITIS, GINGIVAL HYPERPIGMENTATION, SCALLOPED TONGUE, ACID PEPTIC DISEASE, ENTEROCHROMAFFIN CELL HYPERPLASIA, NEONATAL GASTROINTESTINAL DISORDER, GASTRITIS ALCOHOLIC, PSEUDOACHALASIA, MALIGNANT BOWEL OBSTRUCTION, OBSTRUCTIVE PANCREATITIS, INTESTINAL CALCIFICATION, SCIATIC HERNIA, GASTROINTESTINAL SCARRING, ANORECTAL ULCER, ACUTE HAEMORRHAGIC ULCERATIVE COLITIS, AUTOIMMUNE ENTEROPATHY, HIATUS HERNIA, OBSTRUCTIVE, GASTROOESOPHAGEAL HETEROTOPIA, GALLSTONE ILEUS, STRANGULATED UMBILICAL HERNIA, SEGMENTAL DIVERTICULAR COLITIS, NEONATAL INTESTINAL OBSTRUCTION, INGUINAL HERNIA PERFORATION, URINARY ASCITES, OBSTRUCTIVE DEFAECATION, RETROPERITONEAL EFFUSION, INFANTILE DIARRHOEA, GASTRIC PNEUMATOSIS, GASTROINTESTINAL POLYP, INTRINSIC FACTOR DEFICIENCY, BUCCAL POLYP, OESOPHAGEAL MUCOSAL BLISTER, DIAPHRAGMATIC HERNIA, OBSTRUCTIVE, PERITONITIS LUPUS, TOOTH ANKYLOSIS, ABSENT BOWEL MOVEMENT, INTESTINAL VARICES HAEMORRHAGE, OESOPHAGEAL INTRAMURAL HAEMATOMA, OESOPHAGOPLEURAL FISTULA, OMENTAL NECROSIS, ORAL PIGMENTATION, PALATAL ULCER, VISCERAL VENOUS THROMBOSIS, BUCCOGLOSSAL SYNDROME, GASTRODUODENITIS HAEMORRHAGIC, GASTROINTESTINAL MUCOCOELE, PYOSTOMATITIS VEGETANS, PANCREATIC PHLEGMON, RETROPERITONEAL OEDEMA, CHILAIDITI'S SYNDROME, GASTRITIS HYPERTROPHIC, INTESTINAL ATONY, OESOPHAGEAL FIBROSIS, GASTRITIS ALCOHOLIC HAEMORRHAGIC, ANAL FISSURE HAEMORRHAGE, ANASTOMOTIC ULCER PERFORATION, PSEUDODIVERTICULAR DISEASE, INTESTINAL STEATOSIS, GASTRIC ULCER, OBSTRUCTIVE, DUODENAL PAPILLITIS, PREPYLORIC STENOSIS, CHRONIC CHEEK BITING, CANNABINOID HYPEREMESIS SYNDROME, LOWER GASTROINTESTINAL PERFORATION, UPPER GASTROINTESTINAL PERFORATION, GLYCOGENIC ACANTHOSIS, LYMPHOID HYPERPLASIA OF APPENDIX, ORAL MUCOSA ATROPHY, CRYPTITIS, COELIAC ARTERY ANEURYSM, FEMORAL HERNIA, OBSTRUCTIVE, REBOUND ACID HYPERSECRETION, LYMPHOCYTIC OESOPHAGITIS, GASTROPLEURAL FISTULA, MESENTERIC FIBROSIS, DENTAL PARAESTHESIA, SIALOCELE, SIALADENOSIS, ANAL CYST, AORTOENTERIC FISTULA, RETAINED DECIDUOUS TOOTH, STIFF TONGUE, AURICULOTEMPORAL SYNDROME, DIASTEMA, PORTAL HYPERTENSIVE ENTEROPATHY, ORAL HYPERKERATOSIS, MIKULICZ'S DISEASE, MESENTERIC CYST, VISCEROPTOSIS, OBTURATOR HERNIA, GUT FERMENTATION SYNDROME, GASTRIC FIBROSIS, LARGE INTESTINAL ULCER PERFORATION, TONGUE THRUST, URAEMIC GASTROPATHY, PERITONEOCUTANEOUS FISTULA, DUODENAL VARICES, ANORECTAL SENSORY LOSS, TERMINAL ILEITIS, DOLICHOCOLON ACQUIRED, PANCREATIC PSEUDOANEURYSM, INTESTINAL SMOOTH MUSCLE HYPERTROPHY, SPLENIC VEIN ANEURYSM, OVERFLOW DIARRHOEA, LEUKOPLAKIA OESOPHAGEAL, MELANOPLAKIA ORAL, OESOPHAGOMEDIASTINAL FISTULA, ANAL ERYTHEMA, TRANSIENT LINGUAL PAPILLITIS, MESENTERIC ARTERY ANEURYSM, GASTRIC MUCOSAL CALCINOSIS, ENTERIC NEUROPATHY, RECTAL DYSPLASIA, EOSINOPHILIC GASTRITIS, DENTAL DYSAESTHESIA, SALIVARY DUCT STENOSIS, ACUTE OESOPHAGEAL MUCOSAL LESION, ANAL RASH, PELVIC FLOOR HERNIA, PAROTID LIPOMATOSIS, CRONKHITE-CANADA SYNDROME, MALIGNANT GASTROINTESTINAL OBSTRUCTION, ORAL MUCOSAL ROUGHENING, IMMUNE-MEDIATED PANCREATITIS, ORAL LICHENOID REACTION, COLOPATHY, GASTROCOELE, PERITONEAL MESOTHELIAL HYPERPLASIA, STOMACH GRANULOMA, ANAL ECZEMA, OMENTAL OEDEMA, ISCHIATIC HERNIA, SALIVARY GLAND INDURATION, IMMUNE-MEDIATED GASTRITIS, ABDOMINAL WALL OEDEMA, ANAESTHESIA ORAL, MYOCHOSIS, FELINE OESOPHAGUS, WISCHNEWSKY SPOTS, INCREASED INTRAPERITONEAL VOLUME, PULPLESS TOOTH, GASTRIC ULCER HAEMORRHAGE, OBSTRUCTIVE, CRICOPHARYNGEAL ACHALASIA, OESOPHAGEAL STENT STENOSIS, PRECANCEROUS LESION OF DIGESTIVE TRACT, CONTACT STOMATITIS, ACQUIRED ENAMEL HYPOPLASIA, LIP SCAB, ORAL DYSPLASIA, GASTRIC ULCER PERFORATION, OBSTRUCTIVE, MUCOSAL PROLAPSE SYNDROME, ANAL BLISTER, PEPTIC ULCER, OBSTRUCTIVE, CONSTIPATION NEONATAL, ORAL MUCOSAL SCAB, SALIVARY GLAND FISTULA, ANASTOMOTIC ULCER, OBSTRUCTIVE, LICHENOID DYSPLASIA, TONGUE ROUGH, INTESTINAL MUCOSAL TEAR, ORAL PURPURA, PORTAL HYPERTENSIVE COLOPATHY, PANCREATIC PSEUDOCYST HAEMORRHAGE, SUPERIOR MESENTERIC ARTERY DISSECTION, ALLERGIC STOMATITIS, PATHOLOGICAL TOOTH FRACTURE, AMPULLARY POLYP, HAEMORRHAGIC GASTROENTERITIS, OESOPHAGEAL-PULMONARY FISTULA, DUODENAL ULCER, OBSTRUCTIVE, OMENTITIS, SIALODOCHITIS FIBRINOSA, WHITE NIPPLE SIGN, GASTRIC ISCHAEMIA, CORD COLITIS SYNDROME, DIVERTICULAR HERNIA, SUBACUTE PANCREATITIS, RETROPERITONEAL HERNIA, OROFACIAL GRANULOMATOSIS, MICROSCOPIC ENTERITIS, EXCESSIVE GINGIVAL DISPLAY, SCAPHOID ABDOMEN, TONGUE INDURATION, GASTRIC INFARCTION, ORAL MACULE, WALLED-OFF PANCREATIC NECROSIS, RETROPERITONEAL DISORDER, PERIANAL CYST, PERIPANCREATIC VARICES, INTESTINAL BARRIER DYSFUNCTION, COLO-URETHRAL FISTULA, IMMUNE-MEDIATED OESOPHAGITIS, UVULA DEVIATION, INTESTINAL VASCULAR DISORDER, DENTAL ROOT PERFORATION, IDIOPATHIC PANCREATITIS, FOOD PROTEIN-INDUCED ENTEROCOLITIS SYNDROME, INTESTINAL LIPOMATOSIS, OMENTAL HAEMORRHAGE, PALATAL POLYP, DUODENAL BULB DEFORMITY, INTRA-ABDOMINAL CALCIFICATION, ORONASAL FISTULA, GINGIVAL SCAR, STRESS ULCER HAEMORRHAGE, DENTAL ATTRITION, COBBLE STONE TONGUE, PAPILLA OF VATER SCLEROSIS, ABDOMINAL WALL PAIN, SORDES, NICOTINIC STOMATITIS, ABDOMINAL WALL SINUS, OESOPHAGEAL WALL HYPERTROPHY, TOOTH GEMINATION, TOOTH CONCRESCENCE, PALATAL PERFORATION, PERIAPICAL DISEASE, TONGUE EROSION, OMENTAL TORSION, MICRODONTIA, MACRODONTIA, TOOTH FUSION, RANULA, GASTROOESOPHAGEAL REFLUX IN NEONATE, COLLAGENOUS GASTRITIS, OROCUTANEOUS FISTULA, PANCREATIC PSEUDOCYST RUPTURE, CERVICOGENIC DYSPHAGIA, ANAL DISCOMFORT, LOOSE STOOLS, BOWEL SOUNDS ABNORMAL, STOMACH DISCOMFORT, INTESTINAL FUNCTIONAL DISORDER, INTESTINAL HYPERMOTILITY, COLONIC STENOSIS, REFLUX OESOPHAGITIS, MELAENA NEONATAL, REGURGITATION OF FOOD, ACQUIRED PYLORIC STENOSIS, GASTRIC OUTLET OBSTRUCTION, COLONIC POLYP, PARAESTHESIA CIRCUMORAL, MESENTERIC OCCLUSION, ILEITIS, PRURITUS ANI, STOOLS WATERY, PNEUMATOSIS CYSTOIDES INTESTINALIS, INTESTINAL HYPOMOTILITY, BENIGN COLONIC POLYP, ABDOMINAL HAEMATOMA, CAECITIS, TOXIC DILATATION OF COLON, INTESTINAL SPASM, ORAL MUCOSAL DISORDER, POLYP COLORECTAL, LIP SLOUGHING, ORAL SOFT TISSUE DISORDER, COLONIC HAEMORRHAGE, MEGACOLON ACQUIRED, PELVIC PERITONEAL ADHESIONS, PROCTOCOLITIS, SIGMOIDITIS, ORAL MUCOSAL PETECHIAE, COLITIS COLLAGENOUS, DESQUAMATION GINGIVAL, TONGUE BLACK HAIRY, DESQUAMATION MOUTH, COLONIC OBSTRUCTION, HALITOSIS, RECTAL CRAMPS, DIVERTICULUM DUODENAL, VOLVULUS OF BOWEL, TOOTH DECALCIFICATION, GASTRO-INTESTINAL FISTULA, JEJUNITIS, LARGE INTESTINAL STRICTURE, GASTRITIS ATROPHIC, VOMITING NEONATAL, RUPTURED DIVERTICULUM OF COLON, PROCTALGIA FUGAX, GASTROINTESTINAL MUCOSITIS, GASTROENTERITIS NONINFECTIOUS, CAPUT MEDUSAE, PERIPROCTITIS, GASTRIC HYPERPLASIA, TONGUE DESQUAMATION, DUODENAL SCARRING, GASTROOESOPHAGITIS, PANCREAS LIPOMATOSIS, PHARYNGOESOPHAGEAL DIVERTICULUM, ATROPHY OF TONGUE PAPILLAE, BENIGN GASTRIC POLYP, MESENTERIC ATHEROSCLEROSIS, DISTAL ILEAL OBSTRUCTION SYNDROME, CALCIFICATION PANCREATIC DUCT, PAROTID DUCT CYST, SALIVARY GLAND HYPERTROPHY, PERITONEAL EFFUSION, PEPTIC ULCER REACTIVATED, SIDEROPENIC DYSPHAGIA, ANO-RECTAL ULCER, COLONIC ATONY, ANO-RECTAL STENOSIS, DISBACTERIOSIS, OESOPHAGEAL MUCOSAL HYPERPLASIA, ILEORECTAL FISTULA, INTESTINAL GASTRIC METAPLASIA, PERITONEAL MEMBRANE FAILURE, PAROTID GLAND INFLAMMATION, HYPERCEMENTOSIS, PEPTIC ULCER PERFORATION, OBSTRUCTIVE, JEJUNAL FISTULA, PALATITIS, MIKULICZ'S SYNDROME, ANORECTAL SQUAMOUS CELL METAPLASIA, INTESTINAL POLYP HAEMORRHAGE, ORAL LEUKOEDEMA, FLUOROSIS DENTAL, PUDENDAL HERNIA, CREATORRHOEA |
