# Supplementary material for: Gastrointestinal adverse events associated with tirzepatide: A bibliometric and pharmacovigilance analysis
Source: PLoS One. 2026 Mar 27;21(3):e0344289. doi: 10.1371/journal.pone.0344289 (PMC13028446; doi:10.1371/journal.pone.0344289)
Supplement: S3 Table — (DOCX) [file pone.0344289.s004.docx]

## **S3 Table. Generic and brand names of tirzepatide**

| "TIRZEPATIDE", "MOUNJARO TIRZEPATIDE", "CYANOCOBALAMIN TIRZEPATIDE", "MOUNJARO", "OTHER THERAPEUTIC PRODUCTS MOUNJARO", "MOUNJARO ONCE WEEKLY 5MG" "METFORMIN MOUNJARO" |
| --- |
