# Supplementary material for: Gastrointestinal adverse events associated with tirzepatide: A bibliometric and pharmacovigilance analysis
Source: PLoS One. 2026 Mar 27;21(3):e0344289. doi: 10.1371/journal.pone.0344289 (PMC13028446; doi:10.1371/journal.pone.0344289)
Supplement: S4 Table — (DOCX) [file pone.0344289.s005.docx]

## **S4 Table. Contingency table for ROR calculation.** ROR, Reporting Odds Ratio

|  | Tirzepatide | All Other Drugs | Total |
| --- | --- | --- | --- |
| Adverse events | a | c | a+c |
| All other adverse events | b | d | c+d |
| Total | a+b | c+d | a+b+c+d |
| Abbreviation: ROR, Reporting odds ratio.  "a" represents reports where tirzepatide is linked to GI adverse events. "b" shows reports where tirzepatide is linked to non-GI adverse events. "c" indicates reports of other drugs with GI adverse events. "d" refers to reports of other drugs with non-GI adverse events. | | | |
