# Supplementary material for: Gastrointestinal adverse events associated with tirzepatide: A bibliometric and pharmacovigilance analysis
Source: PLoS One. 2026 Mar 27;21(3):e0344289. doi: 10.1371/journal.pone.0344289 (PMC13028446; doi:10.1371/journal.pone.0344289)
Supplement: S5 Table — (DOCX) [file pone.0344289.s006.docx]

## **S5 Table. Number of GIAE cases in patients receiving tirzepatide treatment in FAERS (2022–2024).** GIAE, Gastrointestinal Adverse Event.

| PT | a | Percentage (%) | Cumulative percentage (%) |
| --- | --- | --- | --- |
| Nausea | 4323 | 27.7 | 27.7 |
| Diarrhoea | 1999 | 12.8 | 40.5 |
| Vomiting | 1655 | 10.6 | 51.1 |
| Constipation | 1270 | 8.1 | 59.2 |
| Abdominal Pain Upper | 769 | 4.9 | 64.1 |
| Eructation | 740 | 4.7 | 68.8 |
| Abdominal Discomfort | 610 | 3.9 | 72.7 |
| Dyspepsia | 558 | 3.6 | 76.3 |
| Flatulence | 390 | 2.5 | 78.8 |
| Gastrointestinal Disorder | 388 | 2.5 | 81.3 |
| Abdominal Pain | 386 | 2.5 | 83.8 |
| Abdominal Distension | 383 | 2.5 | 86.3 |
| Pancreatitis | 359 | 2.3 | 88.6 |
| Gastrooesophageal Reflux Disease | 297 | 1.9 | 90.5 |
| Impaired Gastric Emptying | 207 | 1.3 | 91.8 |
| Dry Mouth | 111 | 0.7 | 92.5 |
| Intestinal Obstruction | 59 | 0.4 | 92.9 |
| Pancreatitis Acute | 49 | 0.3 | 93.2 |
| Retching | 48 | 0.3 | 93.5 |
| Frequent Bowel Movements | 42 | 0.3 | 93.8 |
| Gastrointestinal Sounds Abnormal | 42 | 0.3 | 94.1 |
| Gastritis | 35 | 0.2 | 94.3 |
| Gastrointestinal Pain | 34 | 0.2 | 94.5 |
| Small Intestinal Obstruction | 32 | 0.2 | 94.7 |
| Dysphagia | 31 | 0.2 | 94.9 |
| Food Poisoning | 29 | 0.2 | 95.1 |
| Lip Swelling | 28 | 0.2 | 95.3 |
| Vomiting Projectile | 24 | 0.2 | 95.5 |
| Bowel Movement Irregularity | 23 | 0.1 | 95.6 |
| Colitis | 23 | 0.1 | 95.7 |
| Irritable Bowel Syndrome | 22 | 0.1 | 95.8 |
| Swollen Tongue | 19 | 0.1 | 95.9 |
| Ileus | 18 | 0.1 | 96 |
| Haematochezia | 18 | 0.1 | 96.1 |
| Paraesthesia Oral | 17 | 0.1 | 96.2 |
| Pancreatic Disorder | 16 | 0.1 | 96.3 |
| Hypoaesthesia Oral | 15 | 0.1 | 96.4 |
| Regurgitation | 14 | 0.1 | 96.5 |
| Abdominal Rigidity | 12 | 0.1 | 96.6 |
| Faeces Discoloured | 12 | 0.1 | 96.7 |
| Anal Incontinence | 11 | 0.1 | 96.8 |
| Haematemesis | 11 | 0.1 | 96.9 |
| Pancreatitis Necrotising | 11 | 0.1 | 97 |
| Colitis Ischaemic | 10 | 0.1 | 97.1 |
| Faeces Hard | 10 | 0.1 | 97.2 |
| Gastrointestinal Inflammation | 10 | 0.1 | 97.3 |
| Abdominal Pain Lower | 10 | 0.1 | 97.4 |
| Abnormal Faeces | 9 | 0.1 | 97.5 |
| Faecaloma | 9 | 0.1 | 97.6 |
| Toothache | 8 | 0.1 | 97.7 |
| Abdominal Tenderness | 8 | 0.1 | 97.8 |
| Enteritis | 8 | 0.1 | 97.9 |
| Colitis Ulcerative | 8 | 0.1 | 98 |
| Haemorrhoids | 8 | 0.1 | 98.1 |
| Gastrointestinal Motility Disorder | 8 | 0.1 | 98.2 |
| Breath Odour | 8 | 0.1 | 98.3 |
| Pancreatic Cyst | 7 | 0 | 98.3 |
| Rectal Haemorrhage | 7 | 0 | 98.3 |
| Ileus Paralytic | 7 | 0 | 98.3 |
| Diarrhoea Haemorrhagic | 6 | 0 | 98.3 |
| Gastrointestinal Haemorrhage | 6 | 0 | 98.3 |
| Salivary Hypersecretion | 6 | 0 | 98.3 |
| Crohn's Disease | 6 | 0 | 98.3 |
| Gastrointestinal Necrosis | 6 | 0 | 98.3 |
| Tooth Loss | 6 | 0 | 98.3 |
| Gastric Dilatation | 5 | 0 | 98.3 |
| Infrequent Bowel Movements | 5 | 0 | 98.3 |
| Gingival Pain | 5 | 0 | 98.3 |
| Lip Dry | 5 | 0 | 98.3 |
| Coeliac Disease | 5 | 0 | 98.3 |
| Hiatus Hernia | 5 | 0 | 98.3 |
| Glossodynia | 5 | 0 | 98.3 |
| Tongue Discomfort | 4 | 0 | 98.3 |
| Oesophageal Spasm | 4 | 0 | 98.3 |
| Oral Discomfort | 4 | 0 | 98.3 |
| Obstruction Gastric | 4 | 0 | 98.3 |
| Ascites | 4 | 0 | 98.3 |
| Pancreatic Failure | 4 | 0 | 98.3 |
| Obstructive Pancreatitis | 4 | 0 | 98.3 |
| Intestinal Ischaemia | 4 | 0 | 98.3 |
| Stomatitis | 4 | 0 | 98.3 |
| Gastrointestinal Hypomotility | 4 | 0 | 98.3 |
| Aphthous Ulcer | 4 | 0 | 98.3 |
| Volvulus | 4 | 0 | 98.3 |
| Mouth Swelling | 3 | 0 | 98.3 |
| Dyschezia | 3 | 0 | 98.3 |
| Chapped Lips | 3 | 0 | 98.3 |
| Faeces Soft | 3 | 0 | 98.3 |
| Duodenogastric Reflux | 3 | 0 | 98.3 |
| Odynophagia | 3 | 0 | 98.3 |
| Hyperchlorhydria | 3 | 0 | 98.3 |
| Tongue Disorder | 3 | 0 | 98.3 |
| Oesophagitis | 3 | 0 | 98.3 |
| Large Intestinal Obstruction | 3 | 0 | 98.3 |
| Burning Mouth Syndrome | 3 | 0 | 98.3 |
| Functional Gastrointestinal Disorder | 3 | 0 | 98.3 |
| Bezoar | 3 | 0 | 98.3 |
| Epigastric Discomfort | 3 | 0 | 98.3 |
| Proctalgia | 3 | 0 | 98.3 |
| Oral Mucosal Blistering | 3 | 0 | 98.3 |
| Pneumatosis Intestinalis | 3 | 0 | 98.3 |
| Malabsorption | 3 | 0 | 98.3 |
| Intestinal Perforation | 3 | 0 | 98.3 |
| Loose Tooth | 3 | 0 | 98.3 |
| Gastric Ulcer | 3 | 0 | 98.3 |
| Large Intestine Perforation | 3 | 0 | 98.3 |
| Proctitis | 2 | 0 | 98.3 |
| Anal Fissure | 2 | 0 | 98.3 |
| Gastrointestinal Hypermotility | 2 | 0 | 98.3 |
| Change Of Bowel Habit | 2 | 0 | 98.3 |
| Gingival Swelling | 2 | 0 | 98.3 |
| Appendix Disorder | 2 | 0 | 98.3 |
| Lip Discolouration | 2 | 0 | 98.3 |
| Pancreatic Enlargement | 2 | 0 | 98.3 |
| Umbilical Hernia | 2 | 0 | 98.3 |
| Lip Blister | 2 | 0 | 98.3 |
| Melaena | 2 | 0 | 98.3 |
| Intussusception | 2 | 0 | 98.3 |
| Lower Gastrointestinal Haemorrhage | 2 | 0 | 98.3 |
| Stomach Mass | 2 | 0 | 98.3 |
| Diverticulum | 2 | 0 | 98.3 |
| Gastritis Haemorrhagic | 2 | 0 | 98.3 |
| Oral Disorder | 2 | 0 | 98.3 |
| Defaecation Disorder | 2 | 0 | 98.3 |
| Erosive Duodenitis | 2 | 0 | 98.3 |
| Oral Lichen Planus | 2 | 0 | 98.3 |
| Varices Oesophageal | 2 | 0 | 98.3 |
| Enterocolitis | 2 | 0 | 98.3 |
| Gingival Bleeding | 2 | 0 | 98.3 |
| Small Intestinal Perforation | 2 | 0 | 98.3 |
| Oral Pain | 2 | 0 | 98.3 |
| Defaecation Urgency | 2 | 0 | 98.3 |
| Pancreatic Atrophy | 2 | 0 | 98.3 |
| Pancreatitis Chronic | 2 | 0 | 98.3 |
| Dental Caries | 2 | 0 | 98.3 |
| Cheilitis | 2 | 0 | 98.3 |
| Discoloured Vomit | 2 | 0 | 98.3 |
| Oral Mucosal Eruption | 1 | 0 | 98.3 |
| Intra-Abdominal Haemorrhage | 1 | 0 | 98.3 |
| Salivary Gland Enlargement | 1 | 0 | 98.3 |
| Fixed Bowel Loop | 1 | 0 | 98.3 |
| Oedematous Pancreatitis | 1 | 0 | 98.3 |
| Lip Ulceration | 1 | 0 | 98.3 |
| Appendicolith | 1 | 0 | 98.3 |
| Gastrointestinal Tract Irritation | 1 | 0 | 98.3 |
| Reflux Gastritis | 1 | 0 | 98.3 |
| Intestinal Ulcer | 1 | 0 | 98.3 |
| Intestinal Dilatation | 1 | 0 | 98.3 |
| Tongue Oedema | 1 | 0 | 98.3 |
| Gastric Hypomotility | 1 | 0 | 98.3 |
| Gastrointestinal Ulcer | 1 | 0 | 98.3 |
| Oesophageal Obstruction | 1 | 0 | 98.3 |
| Oesophageal Food Impaction | 1 | 0 | 98.3 |
| Anal Prolapse | 1 | 0 | 98.3 |
| Gingival Ulceration | 1 | 0 | 98.3 |
| Abdominal Hernia | 1 | 0 | 98.3 |
| Necrotising Oesophagitis | 1 | 0 | 98.3 |
| Mouth Ulceration | 1 | 0 | 98.3 |
| Omental Infarction | 1 | 0 | 98.3 |
| Duodenitis | 1 | 0 | 98.3 |
| Oesophageal Disorder | 1 | 0 | 98.3 |
| Anorectal Discomfort | 1 | 0 | 98.3 |
| Upper Gastrointestinal Haemorrhage | 1 | 0 | 98.3 |
| Steatorrhoea | 1 | 0 | 98.3 |
| Gastric Haemorrhage | 1 | 0 | 98.3 |
| Dental Discomfort | 1 | 0 | 98.3 |
| Noninfective Sialoadenitis | 1 | 0 | 98.3 |
| Large Intestine Polyp | 1 | 0 | 98.3 |
| Gingival Discomfort | 1 | 0 | 98.3 |
| Saliva Altered | 1 | 0 | 98.3 |
| Pancreatic Mass | 1 | 0 | 98.3 |
| Hyperaesthesia Teeth | 1 | 0 | 98.3 |
| Oesophageal Haemorrhage | 1 | 0 | 98.3 |
| Neurogenic Bowel | 1 | 0 | 98.3 |
| Oesophageal Achalasia | 1 | 0 | 98.3 |
| Pancreatic Fibrosis | 1 | 0 | 98.3 |
| Oesophageal Stenosis | 1 | 0 | 98.3 |
| Glossitis | 1 | 0 | 98.3 |
| Oral Mucosal Exfoliation | 1 | 0 | 98.3 |
| Scalloped Tongue | 1 | 0 | 98.3 |
| Dumping Syndrome | 1 | 0 | 98.3 |
| Epiploic Appendagitis | 1 | 0 | 98.3 |
| Rectal Prolapse | 1 | 0 | 98.3 |
| Proctitis Ulcerative | 1 | 0 | 98.3 |
| Mucous Stools | 1 | 0 | 98.3 |
| Pancreatic Cyst Rupture | 1 | 0 | 98.3 |
| Gastric Perforation | 1 | 0 | 98.3 |
| Haemorrhoids Thrombosed | 1 | 0 | 98.3 |
| Intestinal Infarction | 1 | 0 | 98.3 |
| Vasculitis Gastrointestinal | 1 | 0 | 98.3 |
| Diabetic Gastroparesis | 1 | 0 | 98.3 |
| Tongue Discolouration | 1 | 0 | 98.3 |
| Gastrointestinal Mucosal Disorder | 1 | 0 | 98.3 |
| Duodenal Ulcer | 1 | 0 | 98.3 |
| Lip Exfoliation | 1 | 0 | 98.3 |
| Lip Erythema | 1 | 0 | 98.3 |
| Abdominal Adhesions | 1 | 0 | 98.3 |
| Megacolon | 1 | 0 | 98.3 |
| Oesophageal Pain | 1 | 0 | 98.3 |
| Gastrointestinal Wall Thinning | 1 | 0 | 98.3 |
| Intestinal Mass | 1 | 0 | 98.3 |
| Mesenteric Panniculitis | 1 | 0 | 98.3 |
| Acute Oesophageal Mucosal Lesion | 1 | 0 | 98.3 |
| Retroperitoneal Effusion | 1 | 0 | 98.3 |
| Gastrooesophageal Sphincter Insufficiency | 1 | 0 | 98.3 |
| Oesophageal Ulcer | 1 | 0 | 98.3 |
| Tooth Disorder | 1 | 0 | 98.3 |
| Intra-Abdominal Fluid Collection | 1 | 0 | 98.3 |
| Poor Dental Condition | 1 | 0 | 98.3 |
| Tongue Pruritus | 1 | 0 | 98.3 |
| Internal Hernia | 1 | 0 | 98.3 |
| Lip Pruritus | 1 | 0 | 98.3 |
| Anal Cyst | 1 | 0 | 98.3 |
| Levator Syndrome | 1 | 0 | 98.3 |
| Oesophageal Rupture | 1 | 0 | 98.3 |
| Lip Pain | 1 | 0 | 98.3 |
| Abbreviation: PT, Preferred Term; FAERS, FDA Adverse Event Reporting System; GIAEs, Gastrointestinal Adverse Events. | | | |
