# Supplementary material for: Gastrointestinal adverse events associated with tirzepatide: A bibliometric and pharmacovigilance analysis
Source: PLoS One. 2026 Mar 27;21(3):e0344289. doi: 10.1371/journal.pone.0344289 (PMC13028446; doi:10.1371/journal.pone.0344289)
Supplement: S6 Table — (DOCX) [file pone.0344289.s007.docx]

## **S6 Table.** **RORs of tirzepatide-associated GIAEs at the PT level.** RORs, Reporting Odds Ratio; GIAEs, Gastrointestinal Adverse Events; PT, Preferred Term.

|  | ROR (95%) | | | | |
| --- | --- | --- | --- | --- | --- |
| gastrointestinal disorder (PT) | Overall | Male | Female | ≥65 | <65 |
| Nausea | 5.07 (4.91 - 5.23) | 5.92 (5.48 - 6.39) | 4.07 (3.91 - 4.22) | 4.48 (4.09 - 4.91) | 3.91 (3.74 - 4.09) |
| Diarrhoea | 2.34 (2.24 - 2.45) | 3.35 (3.07 - 3.66) | 1.66 (1.56 - 1.76) | 2.11 (1.89 - 2.36) | 1.73 (1.61 - 1.86) |
| Vomiting | 3.23 (3.08 - 3.4) | 3.86 (3.44 - 4.33) | 2.48 (2.33 - 2.63) | 2.88 (2.49 - 3.33) | 2.11 (1.96 - 2.28) |
| Constipation | 4.72 (4.46 - 5) | 5.37 (4.76 - 6.07) | 3.93 (3.66 - 4.23) | 4.06 (3.54 - 4.67) | 4.42 (4.05 - 4.83) |
| Abdominal Pain Upper | 3.18 (2.96 - 3.42) | 4.64 (3.99 - 5.4) | 2.38 (2.17 - 2.61) | 3.64 (3.02 - 4.39) | 2.43 (2.19 - 2.7) |
| Eructation | 44.65 (41.07 - 48.55) | 57.27 (48.26 - 67.96) | 31.66 (28.31 - 35.42) | 34.1 (27.26 - 42.67) | 38.17 (33.45 - 43.56) |
| Abdominal Discomfort | 2.63 (2.43 - 2.85) | 4.5 (3.84 - 5.27) | 1.93 (1.73 - 2.14) | 3.72 (3.05 - 4.53) | 2.01 (1.77 - 2.27) |
| Dyspepsia | 5.05 (4.64 - 5.5) | 6.85 (5.7 - 8.24) | 3.78 (3.4 - 4.22) | 7.21 (5.9 - 8.81) | 3.59 (3.16 - 4.08) |
| Flatulence | 6.28 (5.67 - 6.96) | 9.07 (7.52 - 10.95) | 4.38 (3.79 - 5.05) | 7.56 (5.99 - 9.54) | 5.11 (4.36 - 6) |
| Gastrointestinal Disorder | 2.88 (2.6 - 3.18) | 4.02 (3.26 - 4.97) | 1.78 (1.53 - 2.06) | 2.01 (1.38 - 2.91) | NA |
| Abdominal Pain | 1.4 (1.27 - 1.55) | 2.26 (1.88 - 2.72) | NA | 1.77 (1.37 - 2.27) | NA |
| Abdominal Distension | 3.19 (2.88 - 3.53) | 5.05 (4.17 - 6.13) | 2.44 (2.14 - 2.79) | 4.78 (3.78 - 6.03) | 2.58 (2.23 - 2.98) |
| Pancreatitis | 8.07 (7.25 - 8.98) | 7.85 (6.26 - 9.85) | 5.96 (5.09 - 6.97) | 4.19 (2.72 - 6.46) | 3.91 (3.22 - 4.75) |
| Gastrooesophageal Reflux Disease | 3.35 (2.99 - 3.76) | 4.24 (3.3 - 5.44) | 2.59 (2.24 - 3) | 4.73 (3.59 - 6.23) | 2.65 (2.22 - 3.16) |
| Impaired Gastric Emptying | 18.41 (15.92 - 21.3) | 23.03 (15.99 - 33.17) | 11.63 (9.59 - 14.11) | 14.17 (8.25 - 24.34) | 9.11 (7.07 - 11.74) |
| Dry Mouth | 1.22 (1.01 - 1.47) | NA | NA | NA | NA |
| Pancreatitis Acute | 2.17 (1.63 - 2.87) | 3.46 (2.25 - 5.33) | 1.72 (1.13 - 2.62) | NA | 1.54 (1.06 - 2.24) |
| Retching | 2.07 (1.56 - 2.75) | 2.57 (1.33 - 4.97) | 1.78 (1.26 - 2.52) | 3.98 (2.3 - 6.89) | NA |
| Gastrointestinal Sounds Abnormal | 6.15 (4.51 - 8.38) | 7.48 (4.1 - 13.65) | 4.28 (2.76 - 6.62) | 12.12 (6.41 - 22.92) | 4.34 (2.63 - 7.17) |
| Gastrointestinal Pain | 2.32 (1.65 - 3.25) | 4.46 (2.52 - 7.9) | NA | 4.61 (2.38 - 8.91) | NA |
| Small Intestinal Obstruction | 2.52 (1.77 - 3.57) | 6.98 (4.47 - 10.9) | NA | 2.84 (1.18 - 6.87) | NA |
| Food Poisoning | 3.37 (2.33 - 4.88) | 4.61 (2.29 - 9.29) | 2.86 (1.79 - 4.56) | 4.34 (1.61 - 11.69) | 2.99 (1.84 - 4.85) |
| Vomiting Projectile | 8.15 (5.4 - 12.31) | NA | 8.51 (5.51 - 13.15) | 11.16 (4.09 - 30.49) | 6.26 (3.64 - 10.76) |
| Bowel Movement Irregularity | 1.74 (1.15 - 2.63) | 4.44 (2.56 - 7.69) | NA | 2.48 (1.03 - 6) | NA |
| Pancreatic Disorder | 3 (1.83 - 4.92) | NA | NA | NA | 2.65 (1.18 - 5.98) |
| Regurgitation | 3.77 (2.22 - 6.42) | 7.18 (3.18 - 16.21) | 3.18 (1.57 - 6.43) | 12.72 (5.59 - 28.98) | 2.39 (1.06 - 5.38) |
| Abdominal Rigidity | 3.86 (2.18 - 6.86) | 5.34 (1.7 - 16.8) | NA | NA | NA |
| Pancreatitis Necrotising | 3.81 (2.09 - 6.95) | 5.7 (2.35 - 13.86) | 2.83 (1.05 - 7.65) | NA | 3.08 (1.45 - 6.55) |
| Breath Odour | 3.65 (1.81 - 7.36) | NA | 3.81 (1.56 - 9.3) | NA | NA |
| Pancreatic Cyst | 3.58 (1.69 - 7.58) | NA | 3.26 (1.34 - 7.95) | NA | 4.1 (1.29 - 13.06) |
| Gastrointestinal Necrosis | 2.44 (1.09 - 5.48) | NA | 4.16 (1.84 - 9.41) | NA | 3.22 (1.32 - 7.86) |
| Gastric Dilatation | 2.92 (1.2 - 7.09) | NA | 3.12 (1.15 - 8.43) | NA | NA |
| Obstruction Gastric | 2.76 (1.02 - 7.43) | NA | NA | NA | NA |
| Obstructive Pancreatitis | 3.26 (1.21 - 8.79) | NA | NA | NA | NA |
| Gastrointestinal Hypomotility | 3 (1.11 - 8.08) | NA | NA | NA | NA |
| Duodenogastric Reflux | 5.51 (1.73 - 17.49) | NA | NA | NA | 6.84 (2.11 - 22.14) |
| Burning Mouth Syndrome | 4.12 (1.3 - 12.99) | NA | NA | NA | NA |
| Intestinal Obstruction | NA | 2.35 (1.59 - 3.49) | NA | NA | NA |
| Ileus Paralytic | NA | 3.49 (1.44 - 8.44) | NA | NA | NA |
| Faeces Hard | NA | 2.99 (1.12 - 8.03) | NA | 3.16 (1.01 - 9.89) | NA |
| Faecaloma | NA | NA | 2.36 (1.17 - 4.76) | NA | 2.96 (1.4 - 6.29) |
| Frequent Bowel Movements | NA | NA | NA | 2.4 (1.39 - 4.14) | NA |
| Irritable Bowel Syndrome | NA | NA | NA | 2.84 (1.18 - 6.87) | NA |
| Abbreviation: PT, Preferred Term; FAERS, FDA Adverse Event Reporting System; RORs, Reporting odds ratio; CI, Confidential Interval.  NA represents not significant. | | | | | |
