# Supplementary material for: Gastrointestinal adverse events associated with tirzepatide: A bibliometric and pharmacovigilance analysis
Source: PLoS One. 2026 Mar 27;21(3):e0344289. doi: 10.1371/journal.pone.0344289 (PMC13028446; doi:10.1371/journal.pone.0344289)
Supplement: S7 Table — (DOCX) [file pone.0344289.s008.docx]

## **S7 Table.** **IC and PRR of tirzepatide-associated** **GIAEs at the SOC level.** IC, Information Component; PRR, Proportional Reporting Ratio; GIAEs, Gastrointestinal Adverse Events; SOC, System Organ Classes.

|  | a | b | c | d | IC(IC025) | PRR（95%CI） |
| --- | --- | --- | --- | --- | --- | --- |
| Overall | 15601 | 64724 | 825120 | 9646082 | 1.29(1.26) | 2.46 (2.43 to 2.5) |
| Female | 9429 | 46784 | 475907 | 5132178 | 0.97(0.94) | 1.98 (1.94 to 2.02) |
| Male | 3338 | 12372 | 245902 | 3139201 | 1.54(1.48) | 2.92 (2.83 to 3.01) |
| <65 | 6740 | 39259 | 337618 | 3752539 | 0.82(0.78) | 1.78 (1.74 to 1.82) |
| ≥65 | 2140 | 8268 | 195538 | 2098510 | 1.26(1.19) | 2.41 (2.32 to 2.5) |
| Abbreviation: IC, information component; PRR , proportional reporting ratio. | | | | | | |
