# Supplementary material for: Gastrointestinal adverse events associated with tirzepatide: A bibliometric and pharmacovigilance analysis
Source: PLoS One. 2026 Mar 27;21(3):e0344289. doi: 10.1371/journal.pone.0344289 (PMC13028446; doi:10.1371/journal.pone.0344289)
Supplement: S8 Table — (DOCX) [file pone.0344289.s009.docx]

**S8 Table. IC of tirzepatide-associated GIAEs at the PT level.** IC, Information Component; GIAEs, Gastrointestinal Adverse Events; PT, Preferred Term.

|  | IC(IC025) | | | | |
| --- | --- | --- | --- | --- | --- |
| gastrointestinal disorder (PT) | Overall | Male | Female | <65 | ≥65 |
| Nausea | 2.23(2.18) | 2.48(2.35) | 1.92(1.86) | 1.87(1.8) | 2.08(1.93) |
| Diarrhoea | 1.19(1.12) | 1.69(1.55) | 0.71(0.61) | 0.77(0.65) | 1.04(0.85) |
| Vomiting | 1.65(1.57) | 1.9(1.71) | 1.27(1.17) | 1.05(0.93) | 1.49(1.25) |
| Constipation | 2.18(2.09) | 2.37(2.17) | 1.92(1.8) | 2.07(1.93) | 1.97(1.74) |
| Abdominal Pain Upper | 1.64(1.52) | 2.16(1.91) | 1.22(1.07) | 1.25(1.07) | 1.82(1.51) |
| Eructation | 5.03(4.91) | 5.32(5.06) | 4.55(4.39) | 4.69(4.5) | 4.67(4.32) |
| Abdominal Discomfort | 1.37(1.24) | 2.12(1.86) | 0.93(0.76) | 0.98(0.77) | 1.85(1.52) |
| Dyspepsia | 2.28(2.14) | 2.69(2.38) | 1.87(1.69) | 1.79(1.58) | 2.76(2.43) |
| Flatulence | 2.58(2.41) | 3.07(2.76) | 2.07(1.83) | 2.27(2.01) | 2.81(2.42) |
| Gastrointestinal Disorder | 1.5(1.33) | 1.96(1.61) | 0.81(0.57) | 0.3(-0.05) | 0.97(0.34) |
| Abdominal Pain | 0.48(0.31) | 1.15(0.84) | 0.02(-0.21) | -0.42(-0.69) | 0.8(0.38) |
| Abdominal Distension | 1.64(1.47) | 2.28(1.96) | 1.26(1.04) | 1.33(1.09) | 2.19(1.8) |
| Pancreatitis | 2.92(2.75) | 2.86(2.48) | 2.48(2.22) | 1.9(1.58) | 1.94(1.21) |
| Gastrooesophageal Reflux Disease | 1.71(1.52) | 2.02(1.6) | 1.34(1.1) | 1.37(1.08) | 2.16(1.7) |
| Impaired Gastric Emptying | 3.97(3.74) | 4(3.41) | 3.33(3.02) | 2.98(2.57) | 3.23(2.33) |
| Dry Mouth | 0.28(-0.03) | -0.12(-1.1) | 0.13(-0.24) | 0.29(-0.13) | -0.51(-1.65) |
| Pancreatitis Acute | 0.35(-0.08) | 1.19(0.52) | -0.43(-1.14) | -0.81(-1.71) | 0.06(-1.24) |
| Retching | 1.09(0.62) | 1.7(0.97) | 0.75(0.04) | 0.6(-0.03) | 0.52(-1.04) |
| Gastrointestinal Sounds Abnormal | 1.02(0.54) | 1.24(0.1) | 0.81(0.23) | 0.49(-0.3) | 1.82(0.88) |
| Gastrointestinal Pain | -0.24(-0.75) | 0.62(-0.17) | -0.84(-1.61) | -1.1(-1.92) | 1.18(0.24) |
| Small Intestinal Obstruction | 2.48(1.97) | 2.51(1.49) | 1.95(1.22) | 1.93(1.09) | 2.94(1.86) |
| Food Poisoning | 1.17(0.6) | 1.95(0.97) | 0.39(-0.48) | 0.18(-0.9) | 1.93(0.79) |
| Vomiting Projectile | 1.28(0.69) | 2.57(1.82) | -0.08(-1.29) | 0.14(-1) | 1.27(-0.29) |
| Bowel Movement Irregularity | 1.67(1.05) | 1.91(0.7) | 1.42(0.63) | 1.47(0.65) | 1.65(-0.12) |
| Pancreatic Disorder | 2.76(2.08) | 1.44(-1.15) | 2.78(2.07) | 2.34(1.44) | 2.36(0.59) |
| Regurgitation | 0.77(0.07) | 1.96(1.02) | -0.76(-2.32) | -0.19(-1.61) | 1.12(-0.44) |
| Abdominal Rigidity | -0.47(-1.18) | -0.08(-2.67) | -0.7(-1.49) | -1.13(-2.21) | 1.27(-0.29) |
| Pancreatitis Necrotising | 1.48(0.64) | 1.14(-0.93) | 0.59(-0.83) | 1.21(-0.21) | NA |
| Breath Odour | 1.76(0.86) | 2.26(0.84) | 1.47(0.26) | 1.09(-0.33) | 2.71(1.29) |
| Pancreatic Cyst | 1.77(0.79) | 1.71(-0.36) | 0.91(-0.65) | 0.59(-1.48) | 1.42(-1.17) |
| Gastrointestinal Necrosis | 1.74(0.72) | 1.98(0.42) | 1.21(-0.56) | 1.41(0.11) | 0.71(-3.07) |
| Gastric Dilatation | 0.68(-0.4) | 1.28(-0.49) | 0.5(-0.92) | 0.33(-1.44) | 1.26(-0.81) |
| Obstruction Gastric | 0.58(-0.56) | -0.46(-4.24) | 1.11(-0.1) | 1.36(0.06) | NA |
| Obstructive Pancreatitis | 1.63(0.42) | 1.3(-1.29) | 1.57(0.01) | 1.06(-1.01) | NA |
| Gastrointestinal Hypomotility | 1.59(0.29) | NA | 1.41(-0.15) | 1.48(-0.59) | NA |
| Duodenogastric Reflux | 0.66(-0.64) | 1.5(-0.06) | -0.59(-4.37) | 0.34(-1.43) | NA |
| Burning Mouth Syndrome | 1.12(-0.3) | NA | 1.71(0.29) | 1.4(-0.16) | NA |
| Intestinal Obstruction | 1.3(-0.26) | 0.86(-2.92) | 1.31(-0.46) | 0.13(-3.65) | 1.7(-0.89) |
| Ileus Paralytic | 1.19(-0.58) | 0.81(-2.97) | 0.09(-3.69) | NA | NA |
| Faeces Hard | 1.36(-0.41) | 0.96(-2.82) | 0.89(-1.7) | 0.38(-3.4) | 1.04(-2.74) |
| Faecaloma | 1.28(-0.49) | NA | 1.14(-1.45) | 0.68(-1.91) | NA |
| Frequent Bowel Movements | 1.72(-0.35) | 2.07(-0.52) | 0.66(-3.12) | 1.86(-0.21) | NA |
| Irritable Bowel Syndrome | 1.49(-0.58) | NA | 0.96(-1.63) | 0.7(-3.08) | NA |
| Abbreviation: PT, Preferred Term; FAERS, FDA Adverse Event Reporting System; IC,Information component; CI, Confidential Interval.  NA represents not significant. | | | | | |
