# Supplementary material for: Gastrointestinal adverse events associated with tirzepatide: A bibliometric and pharmacovigilance analysis
Source: PLoS One. 2026 Mar 27;21(3):e0344289. doi: 10.1371/journal.pone.0344289 (PMC13028446; doi:10.1371/journal.pone.0344289)
Supplement: S9 Table — (DOCX) [file pone.0344289.s010.docx]

## **S9 Table.** PRR tirzepatide-associated GIAEs at the PT level. PRR, Proportional Reporting Ratio; GIAEs, Gastrointestinal Adverse Events; PT, Preferred Term.

|  | PRR (95%CI) | | | | |
| --- | --- | --- | --- | --- | --- |
| gastrointestinal disorder (PT) | Overall | Male | Female | <65 | ≥65 |
| Nausea | 4.85 (4.71 to 5) | 5.70 (5.29 to 6.14) | 3.91 (3.77 to 4.05) | 3.78 (3.62 to 3.94) | 4.32 (3.96 to 4.72) |
| Diarrhoea | 2.31 (2.21 to 2.41) | 3.27 (3.01 to 3.56) | 1.65 (1.55 to 1.75) | 1.72 (1.6 to 1.84) | 2.08 (1.87 to 2.32) |
| Vomiting | 3.19 (3.04 to 3.35) | 3.81 (3.4 to 4.27) | 2.45 (2.30 to 2.60) | 2.09 (1.94 to 2.25) | 2.84 (2.46 to 3.28) |
| Constipation | 4.66 (4.41 to 4.93) | 5.30 (4.7 to 5.97) | 3.89 (3.62 to 4.18) | 4.38 (4.02 to 4.78) | 4.00 (3.49 to 4.58) |
| Abdominal Pain Upper | 3.16 (2.94 to 3.39) | 4.60 (3.96 to 5.34) | 2.37 (2.16 to 2.60) | 2.42 (2.18 to 2.69) | 3.61 (3.00 to 4.35) |
| Eructation | 44.25 (40.72 to 48.08) | 56.67 (47.82 to 67.15) | 31.44 (28.13 to 35.14) | 37.92 (33.25 to 43.24) | 33.82 (27.08 to 42.24) |
| Abdominal Discomfort | 2.62 (2.42 to 2.84) | 4.46 (3.81 to 5.22) | 1.92 (1.73 to 2.13) | 2.00 (1.77 to 2.26) | 3.69 (3.03 to 4.49) |
| Dyspepsia | 5.03 (4.62 to 5.47) | 6.81 (5.67 to 8.18) | 3.77 (3.38 to 4.20) | 3.58 (3.15 to 4.07) | 7.15 (5.86 to 8.72) |
| Flatulence | 6.26 (5.66 to 6.93) | 9.01 (7.48 to 10.86) | 4.36 (3.78 to 5.03) | 5.10 (4.35 to 5.98) | 7.51 (5.96 to 9.46) |
| Gastrointestinal Disorder | 2.87 (2.6 to 3.17) | 4.01 (3.25 to 4.94) | 1.77 (1.53 to 2.05) | 1.23 (1.00 to 1.51) | 2.00 (1.38 to 2.90) |
| Abdominal Pain | 1.40 (1.27 to 1.55) | 2.25 (1.87 to 2.71) | 1.01 (0.88 to 1.16) | 0.74 (0.63 to 0.87) | 1.76 (1.37 to 2.26) |
| Abdominal Distension | 3.18 (2.87 to 3.52) | 5.03 (4.15 to 6.09) | 2.44 (2.14 to 2.79) | 2.57 (2.22 to 2.97) | 4.75 (3.77 to 5.98) |
| Pancreatitis | 8.04 (7.23 to 8.94) | 7.82 (6.24 to 9.80) | 5.94 (5.08 to 6.95) | 3.90 (3.21 to 4.73) | 4.19 (2.72 to 6.45) |
| Gastrooesophageal Reflux Disease | 3.35 (2.99 to 3.76) | 4.23 (3.30 to 5.43) | 2.59 (2.24 to 3.00) | 2.65 (2.22 to 3.16) | 4.71 (3.58 to 6.19) |
| Impaired Gastric Emptying | 18.37 (15.88 to 21.24) | 22.98 (15.97 to 33.08) | 11.61 (9.57 to 14.08) | 9.10 (7.06 to 11.72) | 14.15 (8.25 to 24.28) |
| Dry Mouth | 1.22 (1.01 to 1.47) | 0.92 (0.52 to 1.62) | 1.10 (0.88 to 1.37) | 1.23 (0.96 to 1.58) | 0.69 (0.36 to 1.33) |
| Pancreatitis Acute | 1.28 (0.99 to 1.65) | 2.35 (1.59 to 3.48) | 0.73 (0.48 to 1.11) | 0.56 (0.33 to 0.95) | 1.05 (0.50 to 2.21) |
| Retching | 2.17 (1.64 to 2.88) | 3.46 (2.25 to 5.32) | 1.72 (1.13 to 2.62) | 1.54 (1.06 to 2.24) | 1.51 (0.63 to 3.64) |
| Gastrointestinal Sounds Abnormal | 2.07 (1.56 to 2.75) | 2.57 (1.33 to 4.96) | 1.78 (1.26 to 2.51) | 1.43 (0.9 to 2.28) | 3.97 (2.29 to 6.87) |
| Gastrointestinal Pain | 0.84 (0.62 to 1.14) | 1.57 (0.99 to 2.5) | 0.55 (0.35 to 0.86) | 0.46 (0.29 to 0.74) | 2.40 (1.39 to 4.14) |
| Small Intestinal Obstruction | 6.14 (4.51 to 8.37) | 7.48 (4.1 to 13.64) | 4.28 (2.77 to 6.62) | 4.34 (2.63 to 7.17) | 12.11 (6.41 to 22.88) |
| Food Poisoning | 2.31 (1.65 to 3.24) | 4.46 (2.52 to 7.90) | 1.33 (0.80 to 2.21) | 1.14 (0.61 to 2.13) | 4.60 (2.38 to 8.90) |
| Vomiting Projectile | 2.52 (1.78 to 3.58) | 6.97 (4.47 to 10.88) | 0.94 (0.47 to 1.89) | 1.11 (0.58 to 2.14) | 2.84 (1.18 to 6.86) |
| Bowel Movement Irregularity | 3.37 (2.33 to 4.87) | 4.61 (2.29 to 9.28) | 2.85 (1.78 to 4.55) | 2.99 (1.84 to 4.85) | 4.34 (1.61 to 11.67) |
| Pancreatic Disorder | 8.15 (5.4 to 12.31) | 4.84 (1.19 to 19.65) | 8.51 (5.51 to 13.15) | 6.26 (3.64 to 10.76) | 11.16 (4.09 to 30.47) |
| Regurgitation | 1.74 (1.15 to 2.63) | 4.44 (2.56 to 7.69) | 0.57 (0.24 to 1.37) | 0.86 (0.38 to 1.92) | 2.48 (1.03 to 5.99) |
| Abdominal Rigidity | 0.71 (0.47 to 1.08) | 0.94 (0.23 to 3.77) | 0.61 (0.38 to 0.97) | 0.44 (0.24 to 0.82) | 2.84 (1.18 to 6.86) |
| Pancreatitis Necrotising | 3.00 (1.83 to 4.92) | 2.77 (0.89 to 8.65) | 1.58 (0.71 to 3.54) | 2.65 (1.18 to 5.97) | NA |
| Breath Odour | 3.77 (2.22 to 6.41) | 7.18 (3.18 to 16.19) | 3.18 (1.57 to 6.43) | 2.39 (1.06 to 5.38) | 12.72 (5.59 to 28.96) |
| Pancreatic Cyst | 3.86 (2.17 to 6.85) | 5.34 (1.7 to 16.79) | 2.09 (0.86 to 5.07) | 1.66 (0.53 to 5.20) | 4.69 (1.16 to 19.03) |
| Gastrointestinal Necrosis | 3.81 (2.09 to 6.94) | 5.70 (2.35 to 13.85) | 2.83 (1.05 to 7.64) | 3.08 (1.45 to 6.54) | 2.40 (0.33 to 17.22) |
| Gastric Dilatation | 1.66 (0.89 to 3.10) | 2.99 (1.11 to 8.02) | 1.47 (0.66 to 3.29) | 1.30 (0.48 to 3.49) | 3.16 (1.01 to 9.88) |
| Obstruction Gastric | 1.55 (0.80 to 2.99) | 0.64 (0.09 to 4.56) | 2.36 (1.17 to 4.76) | 2.96 (1.39 to 6.29) | NA |
| Obstructive Pancreatitis | 3.65 (1.81 to 7.37) | 3.92 (0.97 to 15.87) | 3.81 (1.56 to 9.31) | 2.59 (0.82 to 8.16) | NA |
| Gastrointestinal Hypomotility | 3.58 (1.69 to 7.59) | NA | 3.26 (1.34 to 7.94) | 4.10 (1.29 to 13.04) | NA |
| Duodenogastric Reflux | 1.66 (0.79 to 3.50) | 3.49 (1.44 to 8.44) | 0.57 (0.08 to 4.07) | 1.31 (0.49 to 3.52) | NA |
| Burning Mouth Syndrome | 2.44 (1.09 to 5.47) | NA | 4.16 (1.84 to 9.41) | 3.22 (1.32 to 7.86) | NA |
| Intestinal Obstruction | 2.92 (1.2 to 7.08) | 3.08 (0.43 to 22.17) | 3.12 (1.15 to 8.44) | 1.15 (0.16 to 8.27) | 7.60 (1.86 to 31.11) |
| Ileus Paralytic | 2.76 (1.03 to 7.43) | 2.84 (0.39 to 20.42) | 1.10 (0.15 to 7.89) | NA | NA |
| Faeces Hard | 3.26 (1.21 to 8.79) | 3.72 (0.52 to 26.86) | 2.38 (0.59 to 9.67) | 1.53 (0.21 to 11.05) | 4.41 (0.61 to 31.92) |
| Faecaloma | 3 (1.11 to 8.08) | NA | 3.22 (0.79 to 13.16) | 1.91 (0.47 to 7.75) | NA |
| Frequent Bowel Movements | 5.51 (1.74 to 17.49) | 22.68 (5.28 to 97.36) | 2.27 (0.31 to 16.48) | 6.84 (2.11 to 22.13) | NA |
| Irritable Bowel Syndrome | 4.12 (1.31 to 13) | NA | 2.59 (0.64 to 10.54) | 2.40 (0.33 to 17.49) | NA |
| Abbreviation: PT, Preferred Term; FAERS, FDA Adverse Event Reporting System; PRR,proportional reporting ratio; CI, Confidential Interval.  NA represents not significant. | | | | | |
