# Supplementary material for: Gastrointestinal adverse events associated with tirzepatide: A bibliometric and pharmacovigilance analysis
Source: PLoS One. 2026 Mar 27;21(3):e0344289. doi: 10.1371/journal.pone.0344289 (PMC13028446; doi:10.1371/journal.pone.0344289)
Supplement: S10 Table — (DOCX) [file pone.0344289.s011.docx]

## **S10 Table. Time-to-onset analysis of tirzepatide-related GIAEs.** GIAEs, Gastrointestinal Adverse Events.

|  | | | | | |
| --- | --- | --- | --- | --- | --- |
| **Characteristic** | **N ^a^** | **Median (IQR)**  **(days)** | **Scale parameter**  **α (95% CI)** | **Shape parameter**  **β (95% CI)** | **Failure type** |
| Sex |  |  |  |  |  |
| Male | 156 | 13.5(2-51) | 299.06(322.38-464.49) | 0.51(0.38-0.78) | early failure type |
| Female | 357 | 18(2-83) | 447.34(322.38-464.49) | 0.54(0.44-0.70) | early failure type |
| Age |  |  |  |  |  |
| <65 | 320 | 21(2-88.5) | 559.08(322.38-464.49) | 0.54(0.43-0.72) | early failure type |
| ≥65 | 142 | 12(2-42) | 207.68(322.38-464.49) | 0.53(0.40-0.79) | early failure type |
| Overall | 553 | 16(2-70) | 386.96(322.38-464.49) | 0.54(0.45-0.66) | early failure type |
| Abbreviation：GIAEs, Gastrointestinal Adverse Events. IQR, interquartile range  ^a^ Case with valid data for analysis. | | | | | |
