# Supplementary material for: Gastrointestinal adverse events associated with tirzepatide: A bibliometric and pharmacovigilance analysis
Source: PLoS One. 2026 Mar 27;21(3):e0344289. doi: 10.1371/journal.pone.0344289 (PMC13028446; doi:10.1371/journal.pone.0344289)
Supplement: Attachment — Submitted filename: Response to Reviewers_rev1 final.docx [file pone.0344289.s013.docx]

**Response to Editor and Reviewers**

Dear Editor,

We would like to express our sincere gratitude to the reviewers for their insightful comments and constructive suggestions, which have significantly contributed to improving the quality and clarity of our manuscript.

We have carefully revised the manuscript in response to the reviewers’ feedback and are resubmitting it for your kind consideration. All amendments have been highlighted in yellow in the revised version. A detailed, point-by-point response to each comment is provided below this letter.

We hope that the revised manuscript meets the requirements for publication in your journal. We look forward to your favorable response.

With best regards,

**Corresponding Author:**

Kwang Joon Kim, PhD

College of Pharmacy, Chonnam National University, Gwangju 61186, Republic of Korea

Phone: 82 625302929

Fax: 82 625302949

Email: [kjkim0901@jnu.ac.kr](mailto:kjkim0901@jnu.ac.kr)

**Co-corresponding Author**

Kyung-In Joung, PnD

School of AI Healthcare, College of Integrated Health Science, CHA University, Pocheon-si 11160, Republic of Korea

Phone: 82 318509087

Fax: 82 315439917

Email: [jki0515@cha.ac.kr](mailto:jki0515@cha.ac.kr)

We have provided our detailed responses to the reviewers’ comments and suggestions as follows.

**Reviewer #1:**

**Comment 1**: The study was well conducted, appropriately designed, and well explained. The chosen topic is of great scientific interest. The authors present the results impartially. The strengths and limitations of the manuscript are adequately reported. The tables and figures correspond to the presented results and are well addressed in the discussion. The bibliography is up-to-date, although somewhat limited.

**Response 1:** We are deeply grateful for your insightful comments and the time you devoted to reviewing our manuscript. Your careful assessment and constructive suggestions have greatly contributed to improving the overall quality of our work.

**Reviewer #2:**

Thank you for the opportunity to review this manuscript. It's well written and addresses an important issue. However, in my analysis, it raises some concerns, that need to be addressed by the authors.

**Comment 1**: First of all, the manuscript actual focus on gastrointestinal adverse events and not gastrointestinal disorders. Even though that was the selected SOC in the pharmacovigilance analysis. I recommend a change in the title.

**Response 1:** Thank you very much for this important and constructive comment. To better align with the main focus of the study, we have revised the manuscript title as follows: "Gastrointestinal adverse events associated with tirzepatide: a bibliometric and pharmacovigilance analysis"(line 1)

**Comment 2:** The Introduction does not support the relevance and novelty of this study. Lines 49-50, the authors state "... it's use is often associated with gastrointestinal adverse events, such as nausea, vomiting and diarrhea... ", which were the main adverse events also found in this analysis. The fact that other studies using real-word data didn't focus specifically in gastrointestinal adverse events is not justification enough. While studying overall adverse events, if gastrointestinal adverse events were that significant and/or relevant would not appear on the results of those studies?

**Response 2**: Thank you very much for this important and constructive comment. We agree that simply stating that previous studies did not focus on gastrointestinal adverse events (GIAEs) is not sufficient to establish the novelty or necessity of our study. We have therefore revised the Introduction to more clearly articulate why a focused investigation on GIAEs is clinically and scientifically justified.

First, although GIAEs such as nausea, vomiting, and diarrhea have frequently been reported with tirzepatide, they have often been analyzed in aggregated safety outcomes without in-depth exploration. These events, though non-fatal, are among the most common causes of treatment discontinuation and reduced adherence, which has direct implications for real-world therapeutic effectiveness. Despite their frequency, most real-world studies have treated GIAEs as background noise rather than clinically significant events warranting focused evaluation.

Second, tirzepatide has a unique mechanism of action as a dual GIP/GLP-1 receptor agonist. This distinguishes it from previously studied GLP-1 receptor agonists and may lead to a distinct profile of gastrointestinal effects. For example, eructation and gastric emptying delay—highly disproportionate events in our study—have not been systematically characterized in earlier research. This pharmacological distinction provides a mechanistic rationale for examining tirzepatide-specific GIAEs in greater detail.

Third, recent research has shown that GLP-1 receptor agonists exhibit sex- and age-related differences in gastric emptying [Ref. 17], and such variability has not been explored in tirzepatide. Our study aims to fill this gap by assessing GIAE risk across demographic (age, sex), clinical (T2DM vs. obesity), and pharmacological (concomitant medication) subgroups.

Therefore, the novelty of our study lies not merely in isolating GIAEs, but in providing a stratified, real-world safety assessment using complementary approaches: bibliometric mapping to highlight research gaps, and pharmacovigilance analysis to explore subgroup differences. This integrative design enhances our understanding of tirzepatide’s safety profile and addresses the underrepresentation of GIAEs in the literature despite their prevalence in spontaneous reports.

We have reflected these revisions in the Introduction section (lines 53–64) to ensure that the rationale, relevance, and novelty of our research are clearly communicated.

We sincerely appreciate again for your highlighting this critical point, which helped us improve both the clarity and strength of our argument.

**Comment 3**: Other relevant aspect in the Introducion is that no mention is made to the use of tirzepatide in obesity. Is it approved for the clinical use? Therefore, there's no sufficient background for the comparative analyses of T2DM patients vs obese patients.

**Response 3**: We appreciate your insightful comment. You rightly pointed out that our original manuscript did not address the use of tirzepatide in obesity or its current approval status. To provide a more comprehensive background and to strengthen the basis for comparing its use in patients with type 2 diabetes mellitus (T2DM) and those with obesity, we have added relevant information regarding the clinical approval of tirzepatide for obesity and its potential therapeutic role in this population (lines 44–50).

**Comment 4**: In the methods sections, some questions arise that need clarification. First of all, why the single use of Web of Science for the bibliometric analysis? Doesn't that limits the scope of the analysis?

**Response 4**:

First, the topic of "adverse events associated with tirzepatide" spans multiple disciplines—including Clinical Medicine, Pharmacology, Biochemistry, and Public Health—which necessitates the use of a comprehensive and integrated database to ensure the inclusion of relevant literature across various fields.

Second, the WOS Core Collection includes the Science Citation Index Expanded (SCIE), a flagship citation index known for its inclusion of high-quality, peer-reviewed journals.

Third, the database is structured in accordance with Bradford’s Law and Garfield’s Law, enhancing the likelihood that core publications are captured while minimizing the risk of omissions. The exclusive use of the WOS Core Collection is further supported by recent high-quality studies that have employed this dataset alone [10–12].

Nonetheless, we acknowledge the inherent limitation of relying on a single database, which may have resulted in the exclusion of relevant studies from other sources. To address this concern, we included the following statement in the Discussion section (lines 328–332):

*" Firstly, the study relied exclusively on the WOS Core Collection database as its source of research articles, which may have excluded valuable studies published in other databases. However, given the extensive coverage of the WOS Core Collection across most studies, this limitation is unlikely to have significantly impacted the observed trends."*

**Comment 5**: Secondly, the definition of cases and drugs of interest using a System Organ Class leads to too many Preferred Terms, which may disperse the results. However, that was the author's choice. My question is, if the study included cases where tirzepatide was the primary suspect drug, where the data for the comparative analyses came from? From FAERS also? Using what criteria?

**Response 5**: Thank you for your thoughtful question. We appreciate the opportunity to clarify the criteria employed in our comparative analyses.

As described in the Methods section (lines 100–104), we utilized the FDA Adverse Event Reporting System (FAERS) database, which comprises seven interlinked datasets: DEMO (demographics), DRUG (drug data), INDI (indications), OUTC (outcomes), REAC (adverse events coded via MedDRA), PRSR (report source), and THER (therapy dates). In the DRUG file, each drug listed in an adverse event report is assigned a specific role—primary suspect (PS), secondary suspect, concomitant, or interacting drug.

To ensure the accuracy of causal attribution, we restricted our analyses to cases in which **tirzepatide was designated as the primary suspect drug.** This criterion was consistently applied across all stages of analysis, including the selection of gastrointestinal adverse event (GIAE) cases and all subsequent comparative evaluations (e.g., comparisons with other GLP-1 receptor agonists, non-GLP-1 agents, or stratified analyses by indication, sex, or age). Reports in which tirzepatide was classified as a secondary suspect, concomitant, or interacting agent were excluded, as these designations offer a lower level of causal inference reliability [13–15].

We hope this explanation clarifies the methodological rigor underlying our study. We are grateful for your valuable question, which prompted us to improve the transparency and precision of our description.

**Comment 6**: Considering the results analysis and discussion, it lacks a merge between the bibliometric analysis and the pharmacovigilance analysis. Also, data on adverse events are of a different nature between those two sources. Concepts of association, causation are confused in this section.

**Response 6:** Thank you for your insightful comments regarding the integration of findings and the distinction between association and causation. We agree that the initial version of the Discussion section did not sufficiently synthesize the results from the bibliometric and pharmacovigilance analyses.

In response, we revised the Discussion section (lines 263–275) to explicitly integrate the findings from both components of the study. We emphasized how the bibliometric analysis revealed a gap in the literature concerning detailed evaluation of gastrointestinal adverse events (GIAEs), a gap we sought to address using real-world pharmacovigilance data.

Regarding your concern about the potential confusion between association and causation, we have been careful throughout the manuscript to use terminology that clearly distinguishes between the two concepts. Specific sections where this distinction is maintained include lines 23, 189–192, 233, 280–281, 284, and 310. We ensured that the language used avoids causal implication and accurately reflects the observational nature of the data.

We sincerely appreciate your thoughtful feedback, which has significantly contributed to improving the conceptual clarity of our manuscript.

**Comment 7**: Last comment is on the first sentence in the Discussion. "Our bibliometric analysis revealed a sharp increase in research activity on tirzepatide-associated gastrointestinal adverse events (...)". I don't think it did, because the authors also state in the results that "(...) did not appear as an independent theme in the bibliometric map", referring to gastrointestinal adverse events.

**Response 7:** Thank you for your valuable observation. We understand your concern and appreciate the opportunity to clarify the intended meaning of our statement.

Our intention was not to imply that gastrointestinal adverse events (GIAEs) had emerged as a major theme in the bibliometric analysis. Rather, as elaborated more clearly in the revised discussion section, we observed that although research interest in tirzepatide’s overall safety profile has increased substantially following FDA approval, GIAEs were not identified as a distinct thematic cluster in the bibliometric mapping. This observed gap in the literature further reinforces the rationale for our integrated approach.

We appreciate your comment, which enabled us to clarify this important point in the manuscript.

We hope that our integrated approach not only contributes to the growing understanding of tirzepatide’s safety profile but also highlights the importance of patient-specific evaluation in future pharmacovigilance research. We sincerely thank the reviewers for their thoughtful feedback, which has greatly improved the clarity and focus of our work.

**References**

1.	FDA. Available from: https://www.accessdata.fda.gov/scripts/cder/daf/index.cfm?event=overview.process&ApplNo=217806.

2.	FAD. Available from: https://www.accessdata.fda.gov/scripts/cder/daf/index.cfm?event=overview.process&ApplNo=217806.

3.	Aronne LJ, Sattar N, Horn DB, Bays HE, Wharton S, Lin WY, et al. Continued Treatment With Tirzepatide for Maintenance of Weight Reduction in Adults With Obesity: The SURMOUNT-4 Randomized Clinical Trial. Jama. 2024;331(1):38-48. doi: 10.1001/jama.2023.24945. PubMed PMID: 38078870; PubMed Central PMCID: PMCPMC10714284.

4.	Garvey WT, Frias JP, Jastreboff AM, le Roux CW, Sattar N, Aizenberg D, et al. Tirzepatide once weekly for the treatment of obesity in people with type 2 diabetes (SURMOUNT-2): a double-blind, randomised, multicentre, placebo-controlled, phase 3 trial. Lancet. 2023;402(10402):613-26. Epub 20230626. doi: 10.1016/s0140-6736(23)01200-x. PubMed PMID: 37385275.

5.	Caruso I, Di Gioia L, Di Molfetta S, Caporusso M, Cignarelli A, Sorice GP, et al. The real-world safety profile of tirzepatide: pharmacovigilance analysis of the FDA Adverse Event Reporting System (FAERS) database. J Endocrinol Invest. 2024;47(11):2671-8. Epub 20240814. doi: 10.1007/s40618-024-02441-z. PubMed PMID: 39141075; PubMed Central PMCID: PMCPMC11473560.

6.	Ou Y, Cui Z, Lou S, Zhu C, Chen J, Zhou L, et al. Analysis of tirzepatide in the US FDA adverse event reporting system (FAERS): a focus on overall patient population and sex-specific subgroups. Front Pharmacol. 2024;15:1463657. Epub 20241106. doi: 10.3389/fphar.2024.1463657. PubMed PMID: 39568578; PubMed Central PMCID: PMCPMC11576270.

7.	Li J, Xie J, Han Y, Zhang W, Wang Y, Jiang Z. A real-world disproportionality analysis of tirzepatide-related adverse events based on the FDA Adverse Event Reporting System (FAERS) database. Endocr J. 2024. Epub 20241127. doi: 10.1507/endocrj.EJ24-0286. PubMed PMID: 39603650.

8.	Liu L. A real-world data analysis of tirzepatide in the FDA adverse event reporting system (FAERS) database. Front Pharmacol. 2024;15:1397029. Epub 20240607. doi: 10.3389/fphar.2024.1397029. PubMed PMID: 38910884; PubMed Central PMCID: PMCPMC11190169.

9.	Huo Y, Ma M, Liao X. Data mining study on adverse events of tirzepatide based on FAERS database. Expert Opin Drug Saf. 2024:1-9. Epub 20240715. doi: 10.1080/14740338.2024.2376686. PubMed PMID: 39007672.

10.	Zhang L, Zheng H, Jiang ST, Liu YG, Zhang T, Zhang JW, et al. Worldwide research trends on tumor burden and immunotherapy: a bibliometric analysis. Int J Surg. 2024;110(3):1699-710. Epub 20240301. doi: 10.1097/js9.0000000000001022. PubMed PMID: 38181123; PubMed Central PMCID: PMCPMC10942200.

11.	Wu T, Wu Y, Nie K, Yan J, Chen Y, Wang S, et al. Bibliometric analysis and global trends in uterus transplantation. Int J Surg. 2024;110(8):4932-46. Epub 20240801. doi: 10.1097/js9.0000000000001470. PubMed PMID: 38626445; PubMed Central PMCID: PMCPMC11326002.

12.	Wang J, Zhao W, Zhang Z, Liu X, Xie T, Wang L, et al. A Journey of Challenges and Victories: A Bibliometric Worldview of Nanomedicine since the 21st Century. Adv Mater. 2024;36(15):e2308915. Epub 20240117. doi: 10.1002/adma.202308915. PubMed PMID: 38229552.

13.	Rana P, Aleo MD, Wen X, Kogut S. Hepatotoxicity reports in the FDA adverse event reporting system database: A comparison of drugs that cause injury via mitochondrial or other mechanisms. Acta Pharm Sin B. 2021;11(12):3857-68. Epub 20210607. doi: 10.1016/j.apsb.2021.05.028. PubMed PMID: 35024312; PubMed Central PMCID: PMCPMC8727782.

14.	Hua M, Xiong F, Chong S, Zhang Z, Liu Q, Hou J, et al. Abemaciclib increases the risk of venous thromboembolism in breast cancer: Integrate meta-analysis, pharmacovigilance database analysis, and in vitro validation. Cancer Treat Rev. 2024;130:102827. Epub 20240907. doi: 10.1016/j.ctrv.2024.102827. PubMed PMID: 39278067.

15.	Flory JH, Hennessy S, Bailey CJ, Inzucchi SE. Reports of Lactic Acidosis Attributed to Metformin, 2015-2018. Diabetes Care. 2020;43(1):244-6. Epub 20191009. doi: 10.2337/dc19-0923. PubMed PMID: 31597667; PubMed Central PMCID: PMCPMC7011199.
