# Supplementary material for: Gastrointestinal adverse events associated with tirzepatide: A bibliometric and pharmacovigilance analysis
Source: PLoS One. 2026 Mar 27;21(3):e0344289. doi: 10.1371/journal.pone.0344289 (PMC13028446; doi:10.1371/journal.pone.0344289)
Supplement: Attachment — Submitted filename: Response to Reviewers_rev2.docx [file pone.0344289.s014.docx]

**Response to Editor and Reviewers**

Dear Editor,

We would like to express our sincere gratitude to the reviewers for their insightful comments and constructive suggestions, which have significantly contributed to improving the quality and clarity of our manuscript.

We have carefully revised the manuscript in response to the reviewers’ feedback and are resubmitting it for your kind consideration. All amendments have been highlighted in yellow in the revised version. A detailed, point-by-point response to each comment is provided below this letter.

We hope that the revised manuscript meets the requirements for publication in your journal. We look forward to your favorable response.

With best regards,

**Corresponding Author:**

Kwang Joon Kim, PhD

College of Pharmacy, Chonnam National University, Gwangju 61186, Republic of Korea

Phone: 82 625302929

Fax: 82 625302949

Email: [kjkim0901@jnu.ac.kr](mailto:kjkim0901@jnu.ac.kr)

**Co-corresponding Author**

Kyung-In Joung, PnD

School of AI Healthcare, College of Integrated Health Science, CHA University, Pocheon-si 11160, Republic of Korea

Phone: 82 318509087

Fax: 82 315439917

Email: [jki0515@cha.ac.kr](mailto:jki0515@cha.ac.kr)

We have provided our detailed responses to the reviewers’ comments and suggestions as follows.

**Editor #1:**

**Comment 1**: Regarding the selection of the WOSCC core collection, it is essential that you elaborate on the reasons for choosing this database. You may refer to the following articles to support your explanation: PMID: 38229552, 38913439, 40104597, and 38345042.

**Response 1:**

We thank the Editor for this constructive comment. In the revised manuscript, we have expanded the rationale for selecting the Web of Science Core Collection (WoSCC). Specifically, we emphasized its multidisciplinary coverage (e.g., Clinical Medicine, Pharmacology, Biochemistry, and Public Health) and its foundation on Bradford’s Law and Garfield’s Law, which enhances retrieval of core publications. We also cited recent bibliometric studies (PMID: 38229552, 38913439, 40104597, and 38345042) that employed WoSCC as the sole data source, thereby supporting the validity of our methodological choice. The corresponding revisions can be found in the Methods section (lines 83-87).

**Comment 2**: For the analysis of FAERS, a clinical priority assessment should be conducted, and you can consult PMID: 39291217 and 39972562 for relevant methods and perspectives. In addition, when introducing FAERS, it is recommended to incorporate the following literatures: PMID: 40349689 and the article available at https://spj.science.org/doi/10.34133/hds.0325 to enhance the comprehensiveness and authority of your introduction.

**Response 2：**We sincerely thank the editor for this insightful suggestion. In the revised manuscript, we have expanded the description of FAERS by incorporating the recommended references (PMID: 40349689 and https://spj.science.org/doi/10.34133/hds.0325) to enhance the comprehensiveness and authority of the introduction (line 69).

With regard to the clinical prioritization evaluation, we fully agree on its importance in strengthening the interpretation of FAERS analyses (PMID: 39291217; 39972562). The approach described in these references-evaluating emerging signals across dimensions such as number of target events, lower limit of ROR, mortality proportion, criteria for important or designated medical events, and biological plausibility—provides a valuable framework. However, as the FAERS database does not provide sufficiently detailed mortality information for gastrointestinal adverse events associated with tirzepatide, our ability to perform a complete prioritization assessment was limited.

To address this, we have cited the relevant methodological literature (PMID: 39291217; 39972562) in the revised discussion section and explicitly acknowledged this limitation of our study (lines 354-356). We believe this addition helps place our findings into context while highlighting opportunities for future research using data sources that allow for a more comprehensive prioritization analysis.

**Reviewer #3:**

**Comment 1**: The authors should provide more detailed search strategies and screening flowcharts in the bibliometric analysis section. The current methodological description is overly simplified and lacks standardized systematic review processes. Particularly regarding keyword selection and database limitations, the authors could consider expanding the search scope to include other important biomedical databases such as PubMed and Embase to ensure comprehensive literature retrieval. Additionally, the authors should provide detailed descriptions of inclusion and exclusion criteria for literature screening and present inter-rater agreement results from two independent reviewers.

**Response 1:**

We sincerely thank the reviewer for this valuable suggestion. The topic of adverse events associated with tirzepatide spans multiple disciplines, including Clinical Medicine, Pharmacology, Biochemistry, and Public Health, thereby necessitating the use of a comprehensive database to ensure broad coverage. In the present study, we employed the Web of Science (WoS) Core Collection, which is widely recognized for its rigorous indexing standards and extensive inclusion of high-quality peer-reviewed journals. Its structure, grounded in Bradford’s Law and Garfield’s Law, increases the likelihood of capturing core publications while minimizing omissions. This choice is consistent not only with several recent high-quality bibliometric studies [1, 2] but also with a bibliometric analysis published by the academic editor of our manuscript [3-6], who similarly adopted a single-database approach. A detailed rationale for this selection has been provided in the Methods section (lines 83-87). Nevertheless, we fully acknowledge the limitation of relying on a single database, which may have excluded studies indexed exclusively in PubMed, Embase, or other biomedical resources. To address this concern, we have explicitly discussed this issue in the manuscript, while emphasizing that the extensive coverage of WoS makes it unlikely that such omissions have substantially influenced the overall trends observed (lines 341-345).

Our search, conducted on November 18, 2024, used ‘tirzepatide’ and ‘adverse events’ as the primary keywords, supplemented with synonyms to maximize retrieval, which yielded 110 articles and reviews. The complete search strategy and bibliometric records are presented in the Supplementary Materials (S1 Table). It is important to note that bibliometric analysis differs from systematic reviews or meta-analyses. Specifically, bibliometric tools (e.g., VOSviewer, CiteSpace, biblioshiny) automatically remove duplicate and irrelevant records during analysis, thereby minimizing the need for manual screening. For this reason, we did not apply detailed manual inclusion and exclusion criteria or conduct dual independent screening with inter-rater agreement testing, which are methodological steps typically required in systematic reviews. Our approach is consistent with the bibliometric review recommended by the reviewer for our reference (doi:10.1016/j.cpan.2024.12.001).

**Comment 2**: In the FAERS data analysis, the authors should consider adopting more rigorous statistical methods to address potential confounding factors. While the current disproportionality analysis employed reporting odds ratios, it did not consider other algorithms such as IC method, PRR method, etc. Furthermore, the authors should also incorporate the VigiBase database for additional supplementary analysis (doi: 10.1016/j.eclinm.2024.102684).

**Response 2:** Thank you very much for this valuable suggestion. The Reporting Odds Ratio (ROR) is a well-established and widely utilized disproportionality method in pharmacovigilance research. Notably, it has served as the sole analytical approach in a number of high-quality studies, including the article kindly recommended by the reviewer (doi: 10.1016/j.eclinm.2024.102684). Based on its broad acceptance and support in the literature, we selected ROR as the primary analytical approach in the present study. Nevertheless, in order to further enhance the robustness of our results and to fully address the reviewer’s concern, we have additionally conducted complementary analyses using the Information Component (IC) and the Proportional Reporting Ratio (PRR) (lines 139-141). The findings from these supplementary analyses were consistent with our initial results (lines 212-213) and have now been included in the revised supplementary material (S7-9 Table).

With regard to the suggestion of incorporating VigiBase, we sincerely acknowledge its important value as a comprehensive global pharmacovigilance database. Although it was not included in the present study, we agree that integrating high-quality resources such as VigiBase in future research would further enhance the robustness and generalizability of our findings. This point has also been discussed in the limitations section of the revised manuscript (352-360).

**Comment 3**: The authors need to further explore the dose-response relationship and time-dependent characteristics of tirzepatide gastrointestinal adverse events. The current analysis primarily focuses on the frequency and distribution of adverse events but lacks in-depth analysis of event severity, duration, and clinical management strategies. Recent molecular mechanism research on adverse drug reactions shows that gastrointestinal adverse events are often closely related to drug target specificity and signal transduction pathways. The authors should combine tirzepatide's dual GIP/GLP-1 receptor agonist mechanism to deeply analyze the immune microenvironmental basis of its gastrointestinal effects (PMID: 35331128; 37691196; doi: 10.1002/mdr2.70001; 10.1002/mdr2.70007).

**Response 3:**

We sincerely thank the reviewer for this valuable suggestion. We fully agree that investigating the dose-response relationship, time-dependent characteristics, and clinical management strategies of tirzepatide-associated gastrointestinal adverse events would provide important insights. Unfortunately, the FAERS database, as a spontaneous reporting system, does not provide detailed information on drug dosage, treatment duration, event severity, or clinical management. Therefore, such analyses could not be performed within the scope of the present study. To our knowledge, other high-quality studies utilizing the FAERS database have also not been able to address these aspects due to the same inherent data limitations[7-12]. We have carefully acknowledged these limitations in the revised manuscript (lines 345-353) and highlighted that future prospective or real-world cohort studies with more granular data will be essential to address these important aspects (lines 357-365).

Regarding the suggested references (PMID: 35331128; 37691196; doi: 10.1002/mdr2.70001; 10.1002/mdr2.70007), we carefully reviewed them and found that they primarily focus on cancer research rather than the immune microenvironmental mechanisms of gastrointestinal adverse drug reactions. While these works are informative in their respective fields, their relevance to the mechanism of tirzepatide-related gastrointestinal effects may be limited. In the discussion, we discussed the possibility that the dual GIP/GLP-1 receptor agonist mechanism of tirzepatide may represent a plausible pharmacological basis for gastrointestinal adverse events (lines 318-328), supported by relevant literature[13, 14].We believe these clarifications and revisions improve the transparency of our study and appropriately delineate the scope and limitations of FAERS-based analyses.

**Comment 4**: Although the authors conducted subgroup analyses based on gender, age, and indications, they should further consider the impact of other important clinical variables on gastrointestinal adverse event incidence, such as comorbidities, hepatic and renal function status, and racial differences. Particularly in elderly patient populations, polypharmacy and organ function decline may significantly affect drug safety characteristics. The authors could employ multivariate regression analysis or propensity score matching methods to more accurately assess the impact of these confounding factors.

**Response 4:**

We sincerely appreciate the reviewer’s insightful suggestion. We fully agree that incorporating additional clinical variables-such as comorbidities, hepatic and renal function status, racial differences, and polypharmacy in elderly patients-would provide valuable insights into the safety profile of tirzepatide. Unfortunately, the FAERS database, being a spontaneous reporting system, does not capture such detailed clinical information. As a result, it is not feasible to perform multivariate regression analyses, propensity score matching, or other advanced statistical adjustments within the scope of this study. This inherent limitation has also been noted in other high-quality FAERS-based studies[7-12]. We have highlighted this issue in the revised manuscript (lines345-353) and emphasized that future prospective studies or real-world cohort investigations with more comprehensive clinical data will be essential to address these important aspects (lines 357-365).

**Comment 5**: The two analytical components in the current study are relatively independent, lacking deep integration. The authors should establish correlation analyses between literature research hotspots and real-world adverse event reports, exploring differences between academic research focus and actual safety issues occurring in clinical practice. This integrated analysis is of significant importance for guiding future research directions and clinical monitoring priorities. With the widespread application of multi-omics technologies in drug discovery, prediction and mechanistic research of adverse drug reactions also require more systematic methodological support (doi: 10.1016/j.cpan.2024.12.001).

**Response 5:**

We sincerely thank the reviewer for this valuable suggestion. We fully agree that integrating bibliometric analysis with real-world pharmacovigilance data could provide deeper insights into the alignment and discrepancies between academic research priorities and clinical safety signals. In fact, our discussion section has touched upon this aspect: while bibliometric analysis highlighted cardiovascular events as a major research hotspot, our FAERS-based pharmacovigilance assessment revealed gastrointestinal adverse events (GIAEs) as the predominant safety concern reported in clinical practice. This contrast underscores the current research gap regarding tirzepatide-associated GIAEs and highlights the importance of further investigation in this area (lines 276-288).

we agree with the reviewer that future research would benefit from more systematic methodological frameworks. In particular, combining multi-omics approaches, predictive modeling, and real-world safety monitoring has great potential to bridge the gap between research focus and clinical needs.

**Comment 6**: The authors should more comprehensively discuss the inherent limitations of the FAERS database, including reporting bias, missing information, limitations of causal inference, and propose corresponding solutions or explanatory approaches. Additionally, they need to explain how to overcome these limitations in actual clinical applications to improve the practical utility of research results.

**Response 6:**

We sincerely thank the reviewer for this constructive suggestion. We fully agree that a more comprehensive discussion of the inherent limitations of the FAERS database, as well as potential solutions, is essential for improving the interpretability and clinical utility of our findings. In the revised manuscript, we have expanded the discussion of these limitations (lines 345-357). Specifically, we emphasized that FAERS, as a spontaneous reporting system, is subject to underreporting, reporting bias, incomplete data, and potential duplicate records. Moreover, it lacks detailed clinical variables such as drug dosage, treatment duration, event severity, comorbidities, and organ function status, which limits the ability to explore causal relationships or conduct advanced statistical adjustments.

To address these issues, we highlighted several strategies: (1) integrating pharmacovigilance data with prospective cohort studies or electronic health record databases to obtain more granular clinical information; (2) applying complementary methodologies such as active surveillance systems, signal detection algorithms, and mechanistic studies to strengthen causal inference; and (3) encouraging standardized clinical reporting to reduce missing data and reporting bias. From a clinical perspective, overcoming these limitations requires careful interpretation of FAERS signals in conjunction with evidence from randomized controlled trials and real-world cohort studies, thereby improving the applicability of pharmacovigilance findings to patient care.

We have incorporated these points into the revised discussion and conclusion to clarify both the constraints of our study and the potential pathways for enhancing the practical value of FAERS-based research on tirzepatide (lines 357-365).

**References**

1.	Wu T, Wu Y, Nie K, Yan J, Chen Y, Wang S, et al. Bibliometric analysis and global trends in uterus transplantation. Int J Surg. 2024;110(8):4932-46. Epub 20240801. doi: 10.1097/js9.0000000000001470. PubMed PMID: 38626445; PubMed Central PMCID: PMCPMC11326002.

2.	Zhang L, Zheng H, Jiang ST, Liu YG, Zhang T, Zhang JW, et al. Worldwide research trends on tumor burden and immunotherapy: a bibliometric analysis. Int J Surg. 2024;110(3):1699-710. Epub 20240301. doi: 10.1097/js9.0000000000001022. PubMed PMID: 38181123; PubMed Central PMCID: PMCPMC10942200.

3.	Pan D, Wang J, Wang H, Wu S, Guo J, Guo L, et al. Mapping the blueprint of artificial blood vessels research: a bibliometric analysis. Int J Surg. 2025;111(1):1014-31. Epub 20250101. doi: 10.1097/js9.0000000000001877. PubMed PMID: 38913439; PubMed Central PMCID: PMCPMC11745618.

4.	Wang J, Zhao W, Zhang Z, Liu X, Xie T, Wang L, et al. A Journey of Challenges and Victories: A Bibliometric Worldview of Nanomedicine since the 21st Century. Adv Mater. 2024;36(15):e2308915. Epub 20240117. doi: 10.1002/adma.202308915. PubMed PMID: 38229552.

5.	Wang L, Wang J, Zhang Y, Zhang H. Current perspectives and trends of the research on hypertensive nephropathy: a bibliometric analysis from 2000 to 2023. Ren Fail. 2024;46(1):2310122. Epub 20240212. doi: 10.1080/0886022x.2024.2310122. PubMed PMID: 38345042; PubMed Central PMCID: PMCPMC10863539.

6.	Zhang Z, Wang J, Dang S, Liu X, Zhang Y, Zhang H. The worldview of Akkermansia muciniphila, a bibliometric analysis. Front Microbiol. 2025;16:1500893. Epub 20250304. doi: 10.3389/fmicb.2025.1500893. PubMed PMID: 40104597; PubMed Central PMCID: PMCPMC11913835.

7.	Cai X, Chen G, Wang H, Wang L, Hu C. Pharmacovigilance insights into drug-associated venous thromboembolism. Int J Surg. 2025. Epub 20250702. doi: 10.1097/js9.0000000000002931. PubMed PMID: 40607923.

8.	Lin J, Cheng Y, Chen L, Chen M, Shen Z. A real-world analysis of adverse event signals of cognitive and communication disorder in patients treated with Janus kinase inhibitors based on the FAERS database. J Am Acad Dermatol. 2025. Epub 20250807. doi: 10.1016/j.jaad.2025.08.003. PubMed PMID: 40783117.

9.	Wang M, Chen X, Liu Z, Li Z, Zhu Z, Liu S, et al. Exploring potential associations between GLP-1RAs and depressive disorders: a pharmacovigilance study based on FAERS and VigiBase data. EClinicalMedicine. 2025;86:103385. Epub 20250729. doi: 10.1016/j.eclinm.2025.103385. PubMed PMID: 40777864; PubMed Central PMCID: PMCPMC12329256.

10.	Zheng X, Cheng Y, Chen L, Shen Z. A disproportionality analysis of thromboembolic and cardiovascular adverse event signal strength across various JAK-inhibitors based on the FAERS database. J Am Acad Dermatol. 2025;93(3):850-4. Epub 20250524. doi: 10.1016/j.jaad.2025.05.1417. PubMed PMID: 40419222.

11.	Zhu Z, Li Y, Zhu C, Dong Q, Zhang Y, Liu Z, et al. Disproportionality analysis of interstitial lung disease associated with novel antineoplastic agents during breast cancer treatment: a pharmacovigilance study. EClinicalMedicine. 2025;82:103160. Epub 20250318. doi: 10.1016/j.eclinm.2025.103160. PubMed PMID: 40166653; PubMed Central PMCID: PMCPMC11957809.

12.	Zhuang S, Wang B, Wu E, Lin J, Zeng W, Liu M, et al. Adverse cardiac events associated with antibody drug conjugates in cancer patients: a retrospective analysis on the FAERS database and randomized controlled trials. Cardiovasc Res. 2025. Epub 20250602. doi: 10.1093/cvr/cvaf095. PubMed PMID: 40455883.

13.	Samms RJ, Coghlan MP, Sloop KW. How May GIP Enhance the Therapeutic Efficacy of GLP-1? Trends Endocrinol Metab. 2020;31(6):410-21. Epub 20200316. doi: 10.1016/j.tem.2020.02.006. PubMed PMID: 32396843.

14.	Nauck MA, D'Alessio DA. Tirzepatide, a dual GIP/GLP-1 receptor co-agonist for the treatment of type 2 diabetes with unmatched effectiveness regrading glycaemic control and body weight reduction. Cardiovasc Diabetol. 2022;21(1):169. Epub 20220901. doi: 10.1186/s12933-022-01604-7. PubMed PMID: 36050763; PubMed Central PMCID: PMCPMC9438179.
