# Supplementary material for: Gastrointestinal adverse events associated with tirzepatide: A bibliometric and pharmacovigilance analysis
Source: PLoS One. 2026 Mar 27;21(3):e0344289. doi: 10.1371/journal.pone.0344289 (PMC13028446; doi:10.1371/journal.pone.0344289)
Supplement: Attachment — Submitted filename: Response to Reviewers_rev4.docx [file pone.0344289.s016.docx]

**Response to Editor and Reviewers**

Dear Editor,

We would like to express our sincere gratitude to the reviewers for their insightful comments and constructive suggestions, which have significantly contributed to improving the quality and clarity of our manuscript.

We have carefully revised the manuscript in response to the reviewers’ feedback and are resubmitting it for your kind consideration. All amendments have been highlighted in yellow in the revised version. A detailed, point-by-point response to each comment is provided below this letter.

We hope that the revised manuscript meets the requirements for publication in your journal. We look forward to your favorable response.

With best regards,

**Corresponding Author:**

Kwang Joon Kim, PhD

College of Pharmacy, Chonnam National University, Gwangju 61186, Republic of Korea

Phone: 82 625302929

Fax: 82 625302949

Email: [kjkim0901@jnu.ac.kr](mailto:kjkim0901@jnu.ac.kr)

**Co-corresponding Author**

Kyung-In Joung, PnD

School of AI Healthcare, College of Integrated Health Science, CHA University, Pocheon-si 11160, Republic of Korea

Phone: 82 318509087

Fax: 82 315439917

Email: [jki0515@cha.ac.kr](mailto:jki0515@cha.ac.kr)

We have provided our detailed responses to the reviewers’ comments and suggestions as follows.

# Reviewer #3:

## **Comment 1**

Review Comments to the Author

Reviewer #3: The authors have satisfactorily addressed several of the previous concerns. However, regarding the references, I noticed that citations 46, 47, 48, 49, and 51 do not appear to be well-suited for the specific context of this article. I suggest that the authors remove these references to ensure the bibliography is strictly relevant to the study.

**Response 1**

We sincerely thank the reviewer for the careful evaluation of our reference list. Following the reviewer’s suggestion, we have removed them from the revised version and have cited more appropriate references in their place (Lines 276–279).

# Reviewer #4:

## **Comment 1**

Review Comments to the Author

Reviewer #4: (No Response)

**Response 1**

We thank the reviewer for the overall positive evaluation and constructive feedback provided throughout the review process
